# In Vivo Capillary Structure and Blood Cell Flux in the Normal and Diabetic Mouse Eye

**DOI:** 10.1167/iovs.63.2.18

**Published:** 2022-02-09

**Authors:** Kosha Y. Dholakia, Andres Guevara-Torres, Guanping Feng, Derek Power, Jesse Schallek

**Affiliations:** 1 Department of Biomedical Engineering, University of Rochester, Rochester, New York, United States; 2 Center for Visual Science, University of Rochester, Rochester, New York, United States; 3 The Institute of Optics, University of Rochester, Rochester, New York, United States; 4 Flaum Eye Institute, University of Rochester, Rochester, New York, United States; 5 Department of Neuroscience, University of Rochester, Rochester, New York, United States

**Keywords:** adaptive optics, diabetic retinopathy, blood flow, microcirculation, OCT

## Abstract

**Purpose:**

To characterize the early structural and functional changes in the retinal microvasculature in response to hyperglycemia in the Ins2^Akita^ mouse.

**Methods:**

A custom phase-contrast adaptive optics scanning light ophthalmoscope was used to image retinal capillaries of 9 Ins2^Akita^ positive (hyperglycemic) and 9 Ins2^Akita^ negative (euglycemic) mice from postnatal weeks 5 to 18. A 15 kHz point scan was used to image capillaries and measure red blood cell flux at biweekly intervals; measurements were performed manually. Retinal thickness and fundus photos were captured monthly using a commercial scanning laser ophthalmoscope/optical coherence tomography. Retinal thickness was calculated using a custom algorithm. Blood glucose and weight were tracked throughout the duration of the study.

**Results:**

Elevated blood glucose (>250 mg/dL) was observed at 4 to 5 weeks of age in Ins2^Akita^ mice and remained elevated throughout the study, whereas euglycemic littermates maintained normal glucose levels. There was no significant difference in red blood cell flux, capillary anatomy, lumen diameter, or occurrence of stalled capillaries between hyperglycemic and euglycemic mice between postnatal weeks 5 and 18. Hyperglycemic mice had a thinner retina than euglycemic littermates (*p* < 0.001), but retinal thickness did not change with duration of hyperglycemia despite glucose levels that were more than twice times normal.

**Conclusions:**

In early stages of hyperglycemia, retinal microvasculature structure (lumen diameter, capillary anatomy) and function (red blood cell flux, capillary perfusion) were not impaired despite 3 months of chronically elevated blood glucose. These findings suggest that hyperglycemia alone for 3 months does not alter capillary structure or function in profoundly hyperglycemic mice.

Diabetes mellitus is an epidemic that affects more than 135 million people worldwide.^1^^,^^2^ Of the many sequelae of diabetic disease, diabetic retinopathy (DR) is particularly debilitating because it impacts vision earlier in life than other age-dependent eye disease.^3^^–^^6^ DR is the largest cause of working age blindness within developed countries^7^ and, therefore, imparts a loss in societal participation, work abilities, and quality of life due to disability and loss of vision. In the retina, abnormal vascular structure and function associate with reduced contrast sensitivity, visual acuity, and eventually vision loss.^3^^,^^8^ Although the proliferative stages of diabetes are most damaging to vision and the target of many retinal therapies including anti-VEGF treatments and photocoagulation,^9^^–^^11^ it is the early stages of DR that are poorly understood and potentially most useful for diagnosing and treating in response to new therapies in the eye. It is speculated that the hyperglycemic condition imparts both a neural and vascular challenge early in disease, but more information is needed to explore just how early the vascular dysfunction arises. The question is especially pertinent at the level of the microscopic capillaries that mediate metabolite and waste exchange in the neural retina. With a greater understanding of this early natural history of disease, we could better diagnose, treat, and focus therapies by identifying the earliest functional consequences down to the level of the vascular bed that mediates oxygen and metabolite exchange.

Much of our knowledge of vascular changes arises from seminal work in histology that has evaluated vascular beds with high-resolution post mortem tissue. Prolonged hyperglycemia was noted to associate with death of mural cells and subsequently by the weakness of retinal capillaries^12^^,^^13^ followed by the development of microaneurysms and then the growth of weak capillaries, which have the tendency to bleed. And although ex vivo histology has provided very valuable insights about the mechanisms of DR and even tested novel pharmacological treatments,^14^^–^^16^ its nature of study limits observations to a single time point per subject, and does not, for example, provide the ability to track response to therapy that would benefit the individual in personalized medicine regimes. Moreover, histological study does not provide information on the complex dynamics of blood perfusion in the living vascular network.

Recognizing the essential nature of blood flow study in the living eye, numerous imaging modalities have focused on imaging the living vasculature and its response to hyperglycemic challenge.^17^^–^^19^ Of these, optical coherence tomography angiography (OCT-A) has become a powerful new tool that enables the study of microvascular perfusion by examining the motion contrast of microvascular flow.^20^^–^^23^ Unlike fluorescein angiography, no dye is required, meaning the toxicity and off-target effects of fluorescein can be obviated. To date, however, OCT-A provides a map of perfusion that indicates an all-or-none map of perfusion, that is, if blood cells are moving, the vessel is indicated with motion contrast, whereas if it is blocked, the vessel disappears from view. Although useful for diagnostics and response to therapy, there are additional benefits to studying the rate of perfusion in single vessels.

A new suite of high-resolution imaging modalities have emerged in recent years that now allow the detailed study of retinal cells and capillaries which are microscopic and evaded detection with conventional imaging approaches.^18^^,^^24^^–^^27^ This challenge has been solved by correcting the aberrations of the eye with adaptive optics and resolving even the smallest capillaries in the retina.^28^^,^^29^ The study of single capillaries is of heightened interest, because the earliest changes in microvascular perfusion are expected to occur at the capillary level. This finding makes sense when considering the bulk of metabolite exchange and waste removal takes place at the capillary level. And although the study of the structural integrity of these capillaries is essential for understanding the early staging of disease, we could potentially learn far more by studying the early functional consequences in capillaries that go awry early in disease. Toward this end, work has focused to resolve and measure exact measures of red blood cell (RBC) velocity.^18^^,^^26^^,^^30^^,^^31^

RBCs in the healthy retina are constantly in motion and not only need high-resolution imaging, but also fast temporal speeds to facilitate their imaging without motion blur. Traditional raster scanning adaptive optics instruments have a frame rate of about 30 frames per second. This rate is sometimes not enough to track the passage of individual RBCs. Flood illumination systems can be coupled with a camera with a high frame rate, for example 460 frames per second,^27^ but in these approaches phototoxicity can limit the acquisition to a fraction of a second for short wavelength light. Work by the same group has extended acquisition time by using near infrared (NIR) light that is less phototoxic.^32^ In this study, we similarly use NIR light by projecting a 15-kHz line scan across an individual capillary.^33^^,^^34^

This approach allows for the visualization of single RBCs as they pass by the imaging beam. A new metric of RBC flux (cells/s) is now possible. Because the approach does not use dyes and uses nondestructive levels of NIR light, the same capillaries can be tracked over time. In this report, we use this new approach to quantify the changes in RBC flux in the same capillaries of the same mice in conditions of health and hyperglycemia over time.

To model vascular changes at the capillary level during early stage diabetic disease progression, we used noninvasive adaptive optics scanning light ophthalmoscope (AOSLO) imaging. Previously, we have shown that this imaging modality is valid for the determination of RBC flux and capillary lumen diameter, among other metrics.^33^ The nonterminal approach used in this study allows tracking the same animal over the course of the disease and even going back to the same capillary over the progression of hyperglycemia. And although ex vivo histology is greatly informative, it does not capture the complex dynamics of cell loss, such as functional perfusion of the capillaries. This problem is solved by performing in vivo imaging through the optics of the eye.^35^^–^^38^

Here, we apply these techniques to investigate early stage diabetes in an Ins2^Akita^ mouse model that has hyperglycemic onset after juvenile development. This model has been shown to exhibit ocular complications arising from pathological increase in blood glucose.^39^ Vascular dysfunction in the retina has been observed in Ins2^Akita^ mice at 6 months,^40^^–^^42^ but not much is known about its onset. Although this model has been characterized with ex vivo study and conventional resolution in vivo ophthalmoscopy, little is known about the perfusion and microscopic anatomic changes corresponding to the earliest changes associated with hyperglycemia. Here we apply innovative adaptive optics imaging to reveal the anatomy and functional perfusion of the smallest vessels of the retina in the first three months after hyperglycemic onset.

## Methods

Mice were housed at the University of Rochester in compliance with all guidelines from the University Committee on Animal Resources and according to the Association for Research in Vision and Ophthalmology statement for the Use of Animals in Ophthalmic and Vision Research

### Diabetic Model

**Figure 1.**
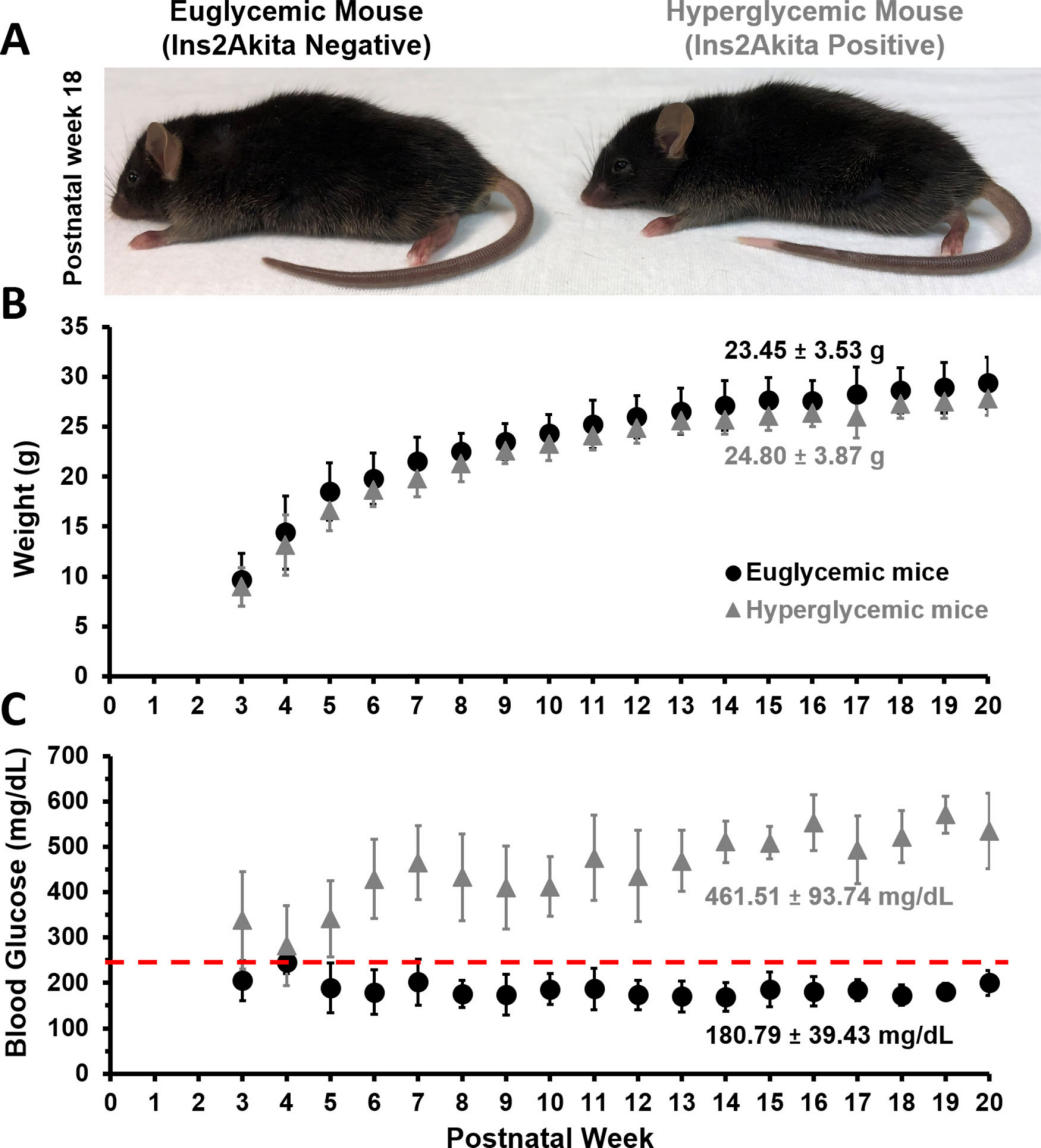
Phenotype appearance, blood glucose and weight measurements for Ins2^Akita^ diabetic mouse model. (**A**) Ins2^Akita^ negative (euglycemic) (*left*) and positive (hyperglycemic) (*right*) mice at postnatal week 18. (**B**) Weight and (**C**) blood glucose measurements are reported for hyperglycemic and euglycemic littermates, average between postnatal weeks 3 to 20 are printed within the figure (mean ± 1 SD). Red line in (**C**) denotes the hyperglycemia cutoff 250 mg/dL.^39^

Heterozygous male Ins2^Akita^-positive mice (Jackson Laboratory, Bar Harbor, ME, Stock No: 003548) were used to model hyperglycemic conditions and Ins2^Akita^-negative littermates served as control animals. In Ins2^Akita^-positive mice, a point mutation of the insulin 2 gene gives rise to misfolded insulin, which accumulates and becomes toxic to the pancreatic beta cells early in age.^43^^,^^44^ The early onset of hyperglycemia models human type 1 diabetes. Starting at postnatal weeks 3 to 5, mice that have misfolded insulin show mild hyperglycemia. which progresses to chronic and sustained blood sugar levels well in excess of 250 mg/dL by postnatal week 5 (Fig. 1C). Because the phenotype is more consistent and severe in male mice, this study was restricted to males. For this study, Ins2^Akita^-positive mice are referred to as hyperglycemic mice and their Ins2^Akita^-negative littermates as euglycemic mice.

### Blood Glucose and Weight Measurements

Blood glucose and weight were recorded weekly upon weaning age (postnatal week 3). A 30G needle was used to puncture the tail tip of the mouse, and blood glucose was sampled by disposable test strips read by a glucose meter (OneTouch Ultra 2, OneTouch, LifeScan, Inc, Milpitas, CA). Mice with consecutive blood glucose measurements exceeding 250 mg/dL were designated as hyperglycemic.^39^ The glucose meter used in this study had a maximum glucose reading cutoff at 600 mg/dL and hence, blood glucose readings higher than that were recorded as 600 mg/dL. Blood glucose and weight were tracked from postnatal weeks 3 to 40 for both groups (Supplementary Tables S1–S4).

### Body Condition Scoring of Hyperglycemic and Euglycemic Mice

General hair coat appearance, activity level, body condition scoring, and body weight are common parameters used to evaluate mouse health.^45^ Visual examination of euglycemic and hyperglycemic mice was done every week during measurement of blood glucose and before AOSLO imaging to determine if the mice showed outward signs of distress.

### Mouse Preparation for Ocular Imaging

Nine hyperglycemic male mice and 10 age-matched euglycemic male littermates were used for this study. Mice were anesthetized using an intraperitoneal injection of ketamine and xylazine (100 mg/kg ketamine, 10 mg/kg xylazine). Anesthesia was then sustained throughout the imaging session by delivering 1% v/v isoflurane with supplemental oxygen through a nose cone. Pupil dilation was achieved with eye drops of 1% tropicamide (Sandoz, Basel, Switzerland) and 2.5% phenylephrine (Akorn, Lake Forest, IL). Mice were placed on a stereotactic stage with five degrees of freedom that allowed both the centration of the pupil and the navigation towards the desired retinal location. A +10 D rigid contact lens with base curvature of 1.6 mm (Advanced Vision Technologies, Lakewood, CO) was placed over the cornea to maintain eye hydration while providing an optical interface for retinal imaging. GenTeal (Alcon Laboratories, Inc, Fort Worth, TX) was administered around the contact lens every 20 minutes during the imaging session to sustain eye hydration. A heat pad was used to maintain mouse body temperature at 37°C.

### AOSLO Imaging

The offset-aperture AOSLO used for these experiments has been described previously.^33^ The sources used are a 796-nm superluminiscent diode (SUPERLUM, Cork, Ireland) for reflectance imaging and a 904-nm laser (QPhotonics, Ann Arbor, MI) for wavefront sensing. The AOSLO is composed of five afocal telescopes that relay the eye's pupil onto a slow galvanometric scanner (25 Hz), a fast resonant scanner (15.4 kHz) and a deformable mirror (ALPAO, Montbonnot-Saint-Martin, France). A Shack–Hartmann wavefront sensor measures the aberrations of the eye and the deformable mirror provides the aberration correction. Reflected 796-nm light from the mouse eye was collected in the phase contrast modality^46^ via photomultiplier tube H7422-50 (Hamamatsu, Shizuoka, Japan). Phase contrast was achieved by placing a 34 Airy Disc Diameter pinhole that was offset laterally by 30 Airy Disc Diameters and axially displaced from the conjugate plane by 14 mm. Cartesian images were captured with either 4.98° × 3.95° or 2.39° × 1.94° field of view (FOV). For recording capillary flux, a 1 D space-time image (line scan) of single file flow capillaries was captured for 20 seconds at a 0.71° FOV using the 15-kHz fast scanner positioned at one location on a vessel.^33^

### Imaging Protocol

**Figure 2.**
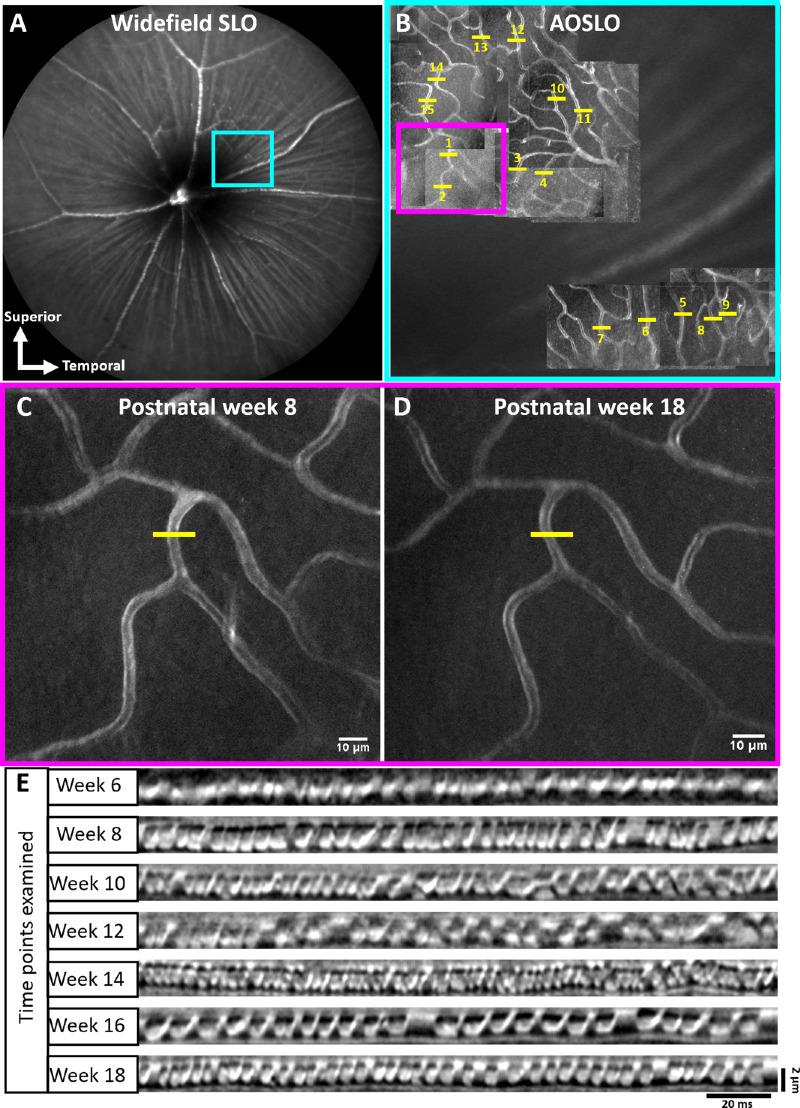
Spatial scheme for longitudinal capillary tracking across 3 months. (**A**) 55° FOV fundus image of mouse retina. Blue box denotes the superior temporal quadrant of the retina targeted for AO imaging. (**B**) Retinal map generated from motion contrast cartesian images acquired during first imaging session (4.98 × 3.95°FOV) (*blue box*). Capillary numbers are denoted in yellow, along with scanner placement (*yellow line*). Motion contrast images of the deep vascular layer targeted for flux measurement (*pink box* in **B**) at postnatal weeks 8 (**C**) and 18 (**D**) (*yellow lines* indicate fast scanner placement). (**E**) Spacetime images (line scans) of the capillary in (**C**) and (**D**) across 3 months.

During the first imaging session, a 55° FOV widefield scanning laser ophthalmoscope (SLO) (Spectralis, Heidelberg Engineering, Heidelberg, Germany) image was used to identify the superior temporal quadrant of the retina between 7° and 22° of eccentricity from optic disc (Fig. 2A). For navigation in this region with the AOSLO, a FOV of 4.98° × 3.95° was used, and capillaries with a vertical configuration in the deepest vascular stratification of the outer plexiform layer were chosen for line scan imaging. Motion contrast images were generated from the cartesian images of the chosen capillaries (captured at 4.98° × 3.95° and 2.39° × 1.94° FOV, registered using a custom software) by calculating standard deviation of each pixel across time. A retinal map was generated from these motion contrast cartesian images after the first imaging session (Fig. 2B) to enable returning to the same capillaries in coming weeks. Imaging sessions lasted between 1 and 2 hours. The power levels for the 796-nm light ranged between 402 and 422 µW and for 904-nm light, 11 µW. RBC flux was measured once every 2 weeks for each mouse in hyperglycemic and euglycemic group from postnatal weeks 5 to 18. OCT thickness was measured every four weeks from postnatal weeks 5 to 20. A timeline of the imaging protocol is shown in Supplementary Figure S1.

### Blood Cell Counting

Space–time images of 1-second duration were generated using a custom MATLAB script from the capillary line scan recordings. A spatiotemporal gaussian filter (σ = 7.5 pixels) was applied to these space–time images to improve image contrast for cell counting. The subsequent second was processed and subject to manual counting of blood cells by recording their locations using the CellCounter plugin (Author: Kurt De Vos, https://imagej.nih.gov/ij/plugins/cell-counter.html) in ImageJ (https://imagej.nih.gov/ij/; Version 1.52i). The centroid of each cell was marked by an expert human grader to indicate its unique position in space and time. Cell counts were processed using a customized MATLAB script to calculate the blood cell flux per second.

Each capillary was assigned a quality index from 1 to 5 in by the human grader based on the image quality and ease of cell counting, with 1 denoting a capillary that could not be visualized and 5 being a high-quality line scan image with distinct blood cells. A detailed rubric is given in Supplementary Figure S2. Capillaries graded 3 and above were counted for blood cell flux analysis.

A randomly selected subset of 38 capillaries with quality index 3 to 5 was assigned to five human graders with varying levels of familiarity with AOSLO line scan imaging for recounting.

### Bland–Altman Analysis

To determine the subjective agreement of manual RBC flux measurements across graders, we performed Bland–Altman analysis. Of the complete data set comprising 988 capillary measurements, we used a computer to randomly select a subset of 38 capillary measurements that met the quality index of 3 or higher (described above). These 38 capillaries were counted by five human graders who were masked to the identity or glycemic state of the mouse. Because there is no established ground truth for RBC flux, the measurements were averaged from each grader and used as ground truth. Each grader's count was compared with the average to produce a standard Bland-Altman plot where ([grader flux – ground truth]/[grader flux + ground truth]/2) is computed for each capillary measurement. The mean bias and ±1.96 standard deviation (limits of agreement) (solid lines) are plotted with 95% confidence intervals calculated using two-sided tolerance factors. This represents the exact parametric confidence intervals for the Bland–Altman limits of agreement.^47^^,^^48^

### Capillary Lumen Diameter Determination

**Figure 3.**
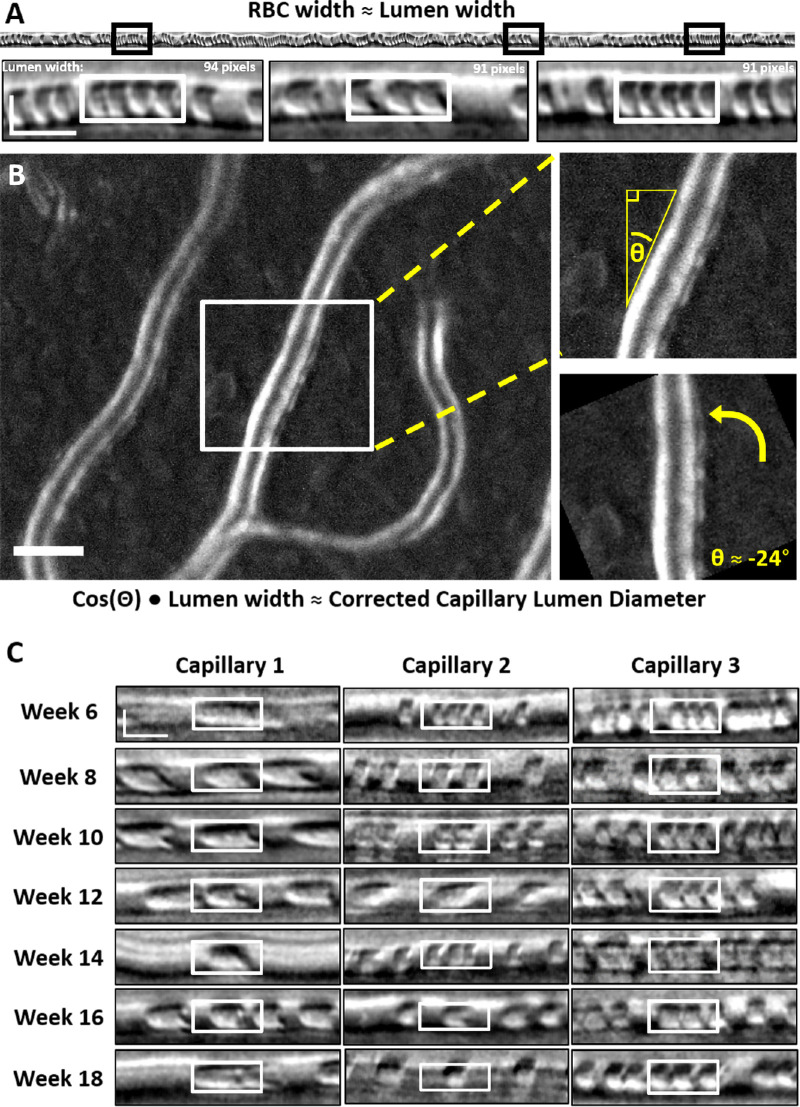
Manual measurement of capillary lumen diameter. (**A**) *Black boxes* denote sample measurement locations from 1-second space-time images. White boxes show sample RBC width measures (in space, vertical axis). Vertical scale bar, 4 µm; horizontal scale bar:, 10 ms. (**B**) Sample motion contrast image (2.39° × 1.94° FOV; *left*) used for manual capillary angle determination. Scale bar, 10 µm. The *white box* represents the crop location for angle determination depicted at right. (**C**) Sample RBC width measurements (*white boxes*) over the study duration for three capillaries, each from a different mouse. Scalebar is same as in (**A**).

Three manual measurements were performed for each line scan image (Fig. 3A, top, black boxes). The width of compressed RBCs was measured manually in ImageJ, using the rectangle tool (Fig. 3A, bottom, white boxes) with a fixed width (15 ms) to determine width of the RBCs within the capillary. RBCs were a requirement for this analysis; stalled capillaries did not yield a diameter. For each time point analyzed, we took into account the increase in image magnification that occurs in the growing mouse eye.^49^ Age-based magnification scaling based on the known angular scanning of the AOSLO is provided in the Supplementary Table S5. For each vessel, three independent measurements were averaged, and the pixel pitch (adjusted for age) was applied to yield the RBC width in micrometers.

Because line scan imaging is conducted in the horizontal dimension, lumen diameters were corrected for the angle between the plane of the line scan and the angle of the capillary intersection at the site of measurement.^34^ The vessel angle was determined from cartesian images (Fig. 3B, left) acquired immediately after each line scan acquisition. The tracked capillary was rotated until the vessel was vertical (Fig. 3B). This rotation corresponds to the vessel angle, θ, relative to the line scan beam and was used to correct the RBC width by the appropriate scaling factor based on *cos(rad(angle))*.

### Age-dependent Analysis of RBC Flux and Lumen Diameter

To characterize the change in RBC flux and lumen diameter with respect to age, we split the mice into two groups representing young (postnatal weeks 5–12) and mature adults (postnatal weeks 13–18).^50^^,^^51^ It is stated that C57BL6 mouse age of 3 to 6 months represents human age of 20 to 30 years.

### Measuring RBC Pulsatility and Eye Displacement Velocity

To determine the potential impact of eye motion contaminating blood cell flux, five randomly chosen 1-second line scan images were evaluated for variations in eye motion, RBC velocity and flux. For RBC velocity, the RBC dwell time (time taken by a cell to pass the imaging beam) was measured manually (ImageJ, box tool) for each cell. RBC instantaneous velocity was calculated as:

$$\mathrm{Vi}\;=\;RBC\;length\;\left(\mu m\right)\;/\;RBC\;dwell\;time\;\left(s\right).$$

Because RBCs are known to compress within capillaries, the dimensions of an RBC were constrained to be 7.25 µm based on the limits of rheology and lysing properties of RBCs previously measured.^33^^,^^52^^–^^54^

To measure eye displacement, the lateral motion of line scan images was computed along the line scan (horizontal) axis using a custom registration algorithm. Measured retinal motion was therefore confined only to motion orthogonal to the vessel. Eye velocity was calculated as:

$$V\;=\;Eye\;displacement\;\left(\mu m\right)\;*\;Scanning\;rate\;\left(Hz\right).$$

Here, the scanning rate is 15.45 kHz. The RBC instantaneous flux was calculated from previously recorded markers placed during manual flux determination. The time between markers was calculated for every pair of consecutive RBCs and for every time point (RBC pair mid-point) along the 1-second line scan, instantaneous flux was calculated as:

Fluxi=scanningrate/numberofscansbetweenmarkers.

### OCT Thickness Measurement

In vivo retinal thickness was measured with a commercial SLO + OCT imaging system (HRA Spectralis, Heidelberg, Germany). The superior–temporal quadrant was centered within the SLO FOV with the optic disc visible for reference. A single NIR reflectance image was captured at weeks 4 to 5 to facilitate longitudinal location consistency using the built-in online eye tracking function (follow-up mode). A three-dimensional retinal cube was imaged two to three times within 30 minutes after the injection of ketamine/xylazine to avoid potential confounds induced by the anesthesia (Feng G, et al. IOVS 2019;60: ARVO E-Abstract 188). Repeat measurements were made every 4 weeks (Supplementary Fig. S1).

The total retinal thickness was measured based on the distance between the vitreous–internal limiting membrane boundary and the outer segment–retinal pigment epithelium boundary (Fig. 10). The vitreous–internal limiting membrane and outer segment–retinal pigment epithelium layers were detected by a custom graph theory dynamic programming segmentation algorithm in a fully automated manner based on the strategy previously reported by Srinivasan et al.^55^ Briefly, each OCT B scan was being treated as a graph. In this graph, the nodes were defined as each pixel in the image, and the edge represented the connection of a pixel (node) with its surrounding 8 pixels. The edges were weighted based on the difference of local image gradient intensity values across two adjacent pixels. Then, the Dijkstra algorithm was used to find a route across the nodes with minimum summed weight (shortest path), which is the position of the specific layer to be segmented. The segmentation algorithm was implemented with MATLAB. The annotated segmentation result was visually examined by an experienced user. Visually incorrect segmentation was uncommon. In rare instances, false segmentations were marked by an expert grader. The total retinal thickness values from the same imaging session were averaged for further analysis.

### Statistical Analysis

All calculations (average, standard deviation, skew, correlation) and statistical tests were performed in Excel. Unless mentioned otherwise, two-tailed unpaired Student's test was used to compare the two populations. Skew was calculated using adjusted Fischer Pearson standardized moment coefficient. Correlation between two variables is displayed in terms of Pearson's coefficient R^2^.

## Results

### Hyperglycemic Mice Have Similar Appearance and Activity Levels as Their Euglycemic Littermates During Early and Mature Adulthood

Elevated blood glucose has the potential to affect mouse health and behavior. However, as a general observation of mouse health, no difference was observed in the grooming, hair coat, or activity levels between hyperglycemic and euglycemic mice (Fig. 1A) for the duration of this study (postnatal weeks 5–18). A body condition score of 3 on a scale of 1 to 5, indicative of the mouse being well-conditioned, was assigned to both groups. Hyperglycemic mice exhibited polydipsia and polyuria by observations of hydropack depletion and cage bedding relative to euglycemic mice. Despite this, the average body weight of hyperglycemic and euglycemic mice was not statistically different during our imaging epoch (Fig. 1B, 23.45 g ± 3.53 vs. 24.80 g ± 3.87; *p* = 0.28). Body weight measurements from postnatal weeks 3 to 40 can be found in Supplementary Tables S1 and S2.

### Hyperglycemic Mice Exhibit Sustained High Blood Glucose Phenotype After Postnatal Week 5

Blood glucose of nine hyperglycemic and euglycemic mice was recorded weekly, starting at postnatal week 3 for the duration of the study. An elevated blood glucose (>250 mg/dL) was observed in hyperglycemic mice as early as postnatal week 3, consistent with reports from Barber et al.^39^ Blood glucose levels became progressively elevated with age for hyperglycemic mice as compared with euglycemic controls and were consistently greater than 250 mg/dL after postnatal week 5 and throughout the duration of the study (461.51 ± 93.74 mg/dL vs. 180.79 ± 39.43 mg/dL; *p* < 0.0001) (Fig. 1C). Blood glucose measurements from postnatal weeks 3 to 40 are listed in Supplementary Tables S3 and S4.

### Capillary Anatomy Remains Intact From Postnatal Weeks 5 to 18

Both wide field SLO and AOSLO imaging allowed us to return to the same locations from postnatal weeks 5 to 18 (Figs. 2A–D). Throughout the 18 weeks of study, we did not observe any anatomic vascular diabetic abnormalities that are common in human diabetic disease, like the development of hairpin turns, aneurysm formation, vascular leakage, or any indication of vascular remodelling^24^^,^^56^ (Supplementary Fig. S8).

### Capillary Lumen Diameter Remains Unchanged Due to Age or Hyperglycemia

**Figure 4.**
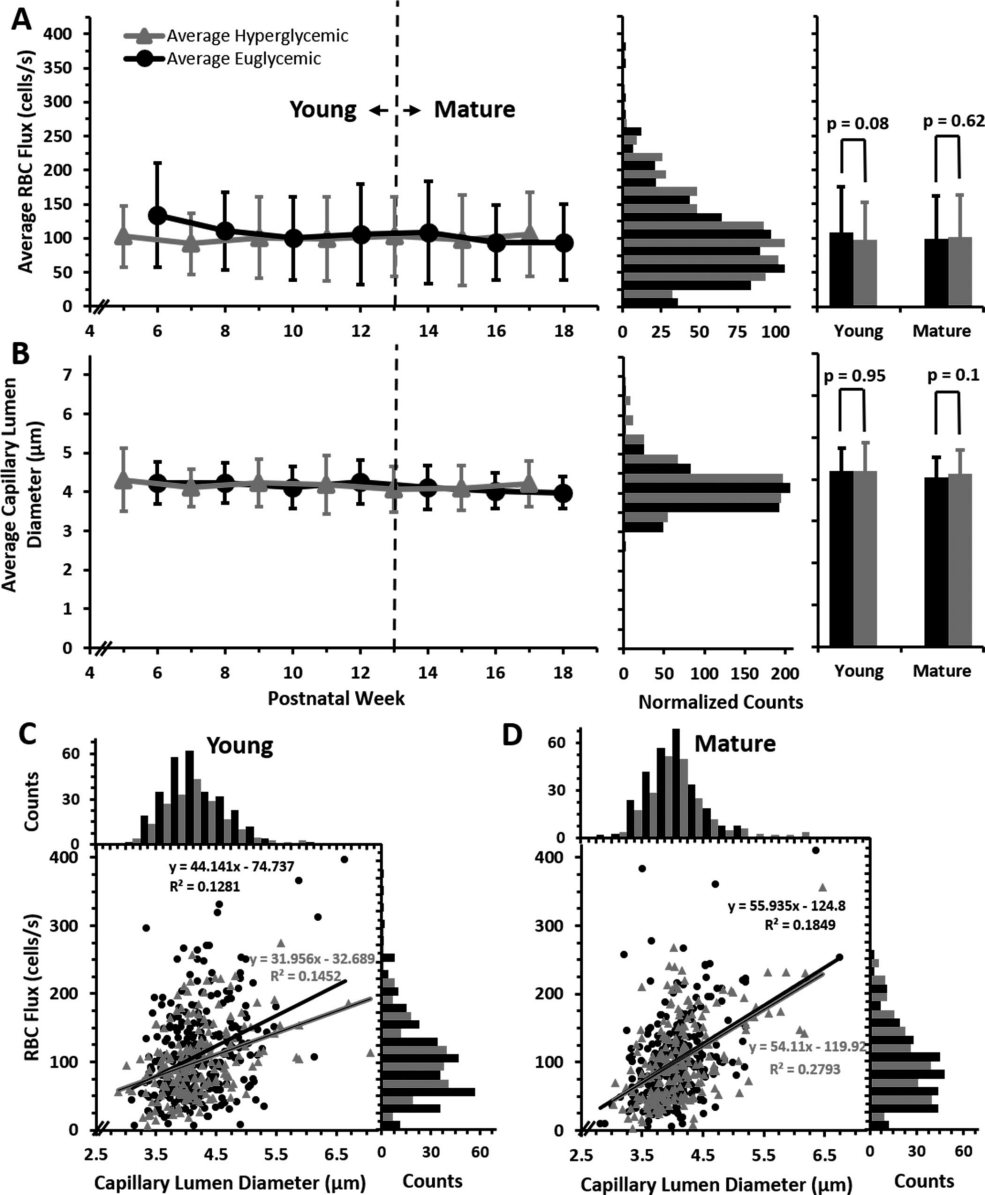
Capillary RBC flux and lumen diameter in euglycemic and hyperglycemic mice. (**A**) Average blood cell flux measured in euglycemic (*n* = 9 mice, 594 measurements from 97 capillaries; *black*) and hyperglycemic mice (*n* = 9 mice, 438 measurements from 86 capillaries; *gray*) plotted from postnatal weeks 5 to 18 (*left*, mean ± 1 SD). Bar graphs (*middle*) display distribution of euglycemic and hyperglycemic flux (bins = 25 cells/s); hyperglycemic data was normalized to total euglycemic counts. Histograms (*right*) display averages for young (<13 weeks) or mature (≥13 weeks) mice (mean ± 1 SD). (**B**) Same series of graphs displayed for capillary lumen diameter measurements (euglycemic, 565 measurements over 97 capillaries; hyperglycemic, 423 measurements over 86 capillaries; *bar graph bins* = 0.5 µm). (**C** and **D**) RBC flux graphed as a function of lumen diameter for euglycemic and hyperglycemic mice in young (*left*) and mature (*right*) groups (0.25 µm bins for lumen diameter and 25 cells/s bins for flux).

In this study, we define capillaries as vessels less than 7 µm in diameter. In mice, RBCs are 6.7 µm in diameter undeformed,^53^^,^^57^ theoretically allowing them to travel through capillaries in single-file. In our data set, we found that, in vessels of this diameter, there was indeed single file flow (Fig. 2E, Figs. 3A, C). We found that capillary lumen diameters were remarkably consistent in the 183 unique capillaries studied (988 measurements) and the erythrocytes traveling within were dominantly parachute shaped (Fig. 3C). This was true for both euglycemic and hyperglycemic mice (97 and 86 capillaries, respectively). In euglycemic mice, lumen diameter ranged from 2.8 to 6.8 µm (mean, 4.1 ± 0.5 µm) and in hyperglycemic, the lumen diameter ranged from 2.9 to 7.0 µm (mean, 4.2 ± 0.6 µm). Lumen diameters measured in this study are similar to those previously reported by our group^33^ (Supplementary Table S6). The weekly average capillary lumen diameters spanned a tight range (4.0–4.3 µm) for the duration of the study (Fig. 4B, left). Lumen diameter distributions for the euglycemic and hyperglycemic data sets were similar; both normally distributed with very little variance (Fig. 4B, middle).

We did not see any significant diameter differences in young and mature groupings of these populations (young, *p* = 0.95; mature, *p* = 0.1) (Fig. 4B, right). Over time and in both populations, we found very little change, indicating that, in the capillaries tracked, both age and elevated blood glucose had no measured effect on lumen diameter.

### Scope and Extent of Capillary Flux Data Set

The data set analyzed here represents the first of its kind to track the same capillaries at biweekly intervals in 18 mice. We evaluated 183 unique capillary segments for blood cell flux (86 for hyperglycemic, 97 for euglycemic). Between 6 and 14 capillaries were tracked in each animal. The total number of counted blood cells was 104,524 cells for postnatal weeks 5 to 18.

### Quality of the Capillary Flux Data Set

As described in the Methods, all capillary scans were subjectively ranked on a 1 to 5 scale based on quality of contrast and features in the data. From 1029 line scans captured (440 for hyperglycemic and 589 for euglycemic mice), 96% of the captured data (95.4% hyperglycemic data, 97.3% euglycemic data) was within the range of 3 to 5, and only 4% of the data was discarded because it did not meet the predefined evaluation criteria (Supplementary Fig. S2).

### Longitudinal Imaging Rate of Success for RBC Flux and Retinal Thickness

Eighteen mice survived throughout the postnatal 5 to 18 week imaging span. One euglycemic mouse died prematurely at week 8 from anesthesia complications and, therefore, those data were excluded. Of the 183 unique capillary segments tracked across 18 mice, all but two capillaries could be tracked at multiple time points (98.9% of data could be followed). Moreover, 86.05% of hyperglycemic capillaries and 100% of euglycemic capillaries were tracked for four of the seven time points. Overall, 19.76% of hyperglycemic and 33.99% of euglycemic capillary segments were tracked for all 7 weeks of imaging (Supplementary Figs. S3–S5).

In tracking the retinal thickness of these 18 mice from postnatal weeks 5 to 20 every 4 weeks, 72 total OCT cubes were acquired successfully (100% success rate). Thirty-six OCT cubes were acquired for hyperglycemic and euglycemic mice each. Our custom algorithm was able to measure retinal thickness without any false segmentation for 68 out of the 72 OCT cubes (94.4% success rate, 34 hyperglycemic and 34 euglycemic retinal thickness measurements)

Factors limiting a 100% success rate for flux imaging were attributed to preparation variables, including transient cataract formation, preparation stability (e.g., length of stable anesthesia permitting recording), and the quality of ocular preparation (contact lens placement, eye hydration, etc.). Variation in image quality and contrast attributed to factors listed above limited a 100% success rate in measuring retinal thickness from the acquired OCT cubes.

### Hyperglycemic Mice Have Capillary Blood Flux Similar to Euglycemic Littermates

Capillary flux in euglycemic and hyperglycemic mice were not statistically different from postnatal weeks 5 to 18, which represent the first 13 weeks of sustained hyperglycemia (102.77 ± 65.12 cells/s for euglycemic and 99.85 ± 58.53 cells/s for hyperglycemic mice) (Fig. 4A, left) (*p* = 0.88). The population of cell flux measurements were normally distributed for each group (Fig. 4A, middle) with a small positive skew (adjusted Fischer Pearson standardized moment coefficient, 0.80 for euglycemic and 0.84 for hyperglycemic mice) and kurtosis (−0.97 for euglycemic mice and −0.86 hyperglycemic mice). In addition to similarities in the average and distribution, the range of the blood flux in capillaries was similar in the two populations (hyperglycemic 0–356.57 cells/s, euglycemic 0–410.08 cells/s).

### Average Blood Flux Remained Consistent as Hyperglycemic and Euglycemic Mice Aged

To further characterize the nuances of age dependency, we split the mice into two groups representing young (postnatal weeks 5–12) and mature adults (postnatal weeks 13–18).^50^^,^^51^

When averaging all measured capillaries, there was no significant difference between the RBC flux of young and mature adult hyperglycemic (*p* = 0.94) (101.48 ± 49.28 cells/s vs. 102.08 ± 55.94 cells/s). In addition, the RBC flux of young and mature euglycemic mice was similar (*p* = 0.41) (106.65 ± 59.63 cells/s vs. 99.67 ± 57.13 cells/s). On comparing hyperglycemic and euglycemic conditions, no difference was observed between young hyperglycemic and euglycemic mice (*p* = 0.95) (101.48 ± 49.28 cells/s vs. 106.65 ± 59.63 cells/s) or between mature hyperglycemic and euglycemic mice (*p* = 0.1) (102.08 ± 55.94 cells/s vs. 99.67 ± 57.13 cells/s) (Fig. 4A, right).

We found little correlation between age and capillary flux in either population, indicating stable measurements over time, but also a consistent vascular perfusion at the capillary level regardless of sustained hyperglycemia throughout 13 weeks. Despite these overall similarities, we now illustrate the heterogeneity in measurements across mice and across capillaries in the same network within a single mouse.

### Despite Similar Lumen Diameter, Blood Cell Flux Is Heterogeneous in Capillaries

An early finding^33^ was that, despite having a similar diameter, RBC flux may be high or low within single capillaries. The current report confirms this finding with a larger data set from more mice and more time points. We also find a poor flux–diameter correlation with the hyperglycemic population. We examine the potential sources of this variability in turn.

### Blood Cell Flux Is Weakly Correlated With Lumen Diameter

A leading hypothesis is that a reduced lumen diameter may reduce RBC flux. Therefore, we measured the capillary lumen diameter in both hyperglycemic and euglycemic mice. We found that, in both populations of mice, lumen diameters were consistent across postnatal weeks 5 to 18 (Fig. 4B). The relationship between flux and diameter was weakly correlated in both hyperglycemic (R^2^ = 0.21, slope = +42.9 cells/s/µm) and euglycemic mice (R^2^ = 0.16, slope = +49.5 cells/s/µm). This finding was true even after classifying the mice into young and mature age ranges (hyperglycemic mice: young, R^2^ = 0.15, slope = 31.9 cells/s/µm; mature, R^2^ = 0.27, slope = +54.1 cells/s/µm; euglycemic mice: young, R^2^ = 0.12, slope = +44.1 cells/s/µm; mature, R^2^ = 0.18, slope = +55.9 cells/s/µm) (Figs. 4C and D), consistent with our earlier study.^33^ The unit (cells/s/µm) describes the expected rate of flux increase for every µm increase in lumen diameter in capillaries 3 to 7 µm, assuming a linear regression.

### Average Flux Measured in Different Mice Across Postnatal Weeks 5 to 18

**Figure 5.**
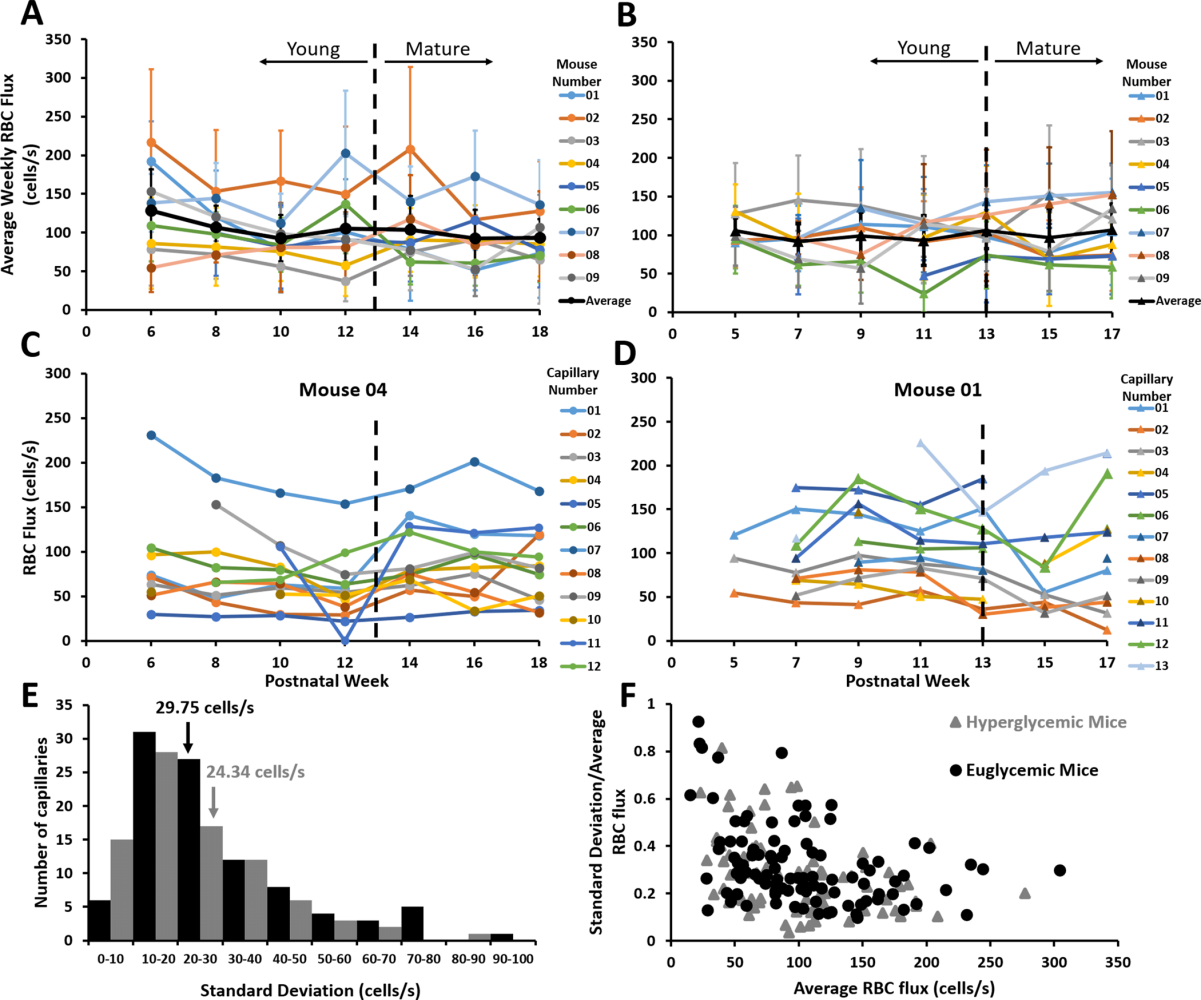
RBC flux distribution for euglycemic and hyperglycemic mice. (**A**) Average RBC flux distribution of nine euglycemic mice measured biweekly across postnatal weeks 6 to 18 (mean ± 1 SD) as a function of postnatal week. The *black line* denotes group average. (**B**) The average RBC flux distribution of nine hyperglycemic mice (mean ± 1 SD) from postnatal weeks 5 to 17. Black line denotes group average. (**C** and **D**) RBC flux for capillaries of a single representative euglycemic (**C**) and hyperglycemic mouse (**D**). Any instance where RBC flux becomes zero refers to a stalled capillary. (**E**) Distribution of standard deviation of hyperglycemic (*grey*) and euglycemic (*black*) capillaries. Inset numbers denote average standard deviation. (**F**) RBC flux standard deviation as a fraction of mean as a function of RBC flux average.

The highest average RBC flux across postnatal weeks 5 to 18 for hyperglycemic mice was 277.11 ± 55.54 cells/s. The highest recorded average RBC flux was 304.25 ± 90.68 cell/s for a euglycemic mouse. The lowest average RBC flux for hyperglycemic mice was 23.2 ± 14.52 cells/s, and it was 14.98 ± 9.20 cells/s for euglycemic mice (Figs. 5A, B).

Despite similar genetics and age, within both the hyperglycemic and the euglycemic groups, some mice had consistently high-flow throughout (e.g., mouse 02 in Fig. 5A and mouse 03 in Fig. 5B), and some mice had consistently low flow in the sampled single file flow capillaries across each imaging time point (e.g., mouse 03 Fig. 5 Aa and mouse 06 Fig. 5B). Other mice had flux that varied from week to week (mouse 0 Fig. 5A and mouse 09 Fig. 5 B). This trend was consistent across young and mature age ranges.

### Range of Observed RBC Flux

The highest RBC flux recorded for a hyperglycemic mouse capillary was 356.58 cells/s. The highest recorded RBC flux in a euglycemic mouse capillary was 410.07 cells/s. The lowest observed flux in each population was 0, which indicated a rare, stalled capillary that was always observed to reperfuse. The lowest, nonstalled flux we measured was 2.7 cells/s (hyperglycemic) and 4.3 cells/s (euglycemic) (Supplementary Figs. S4, S5).

### Flux Consistency in the Same Capillaries from Week to Week

Although the range of RBC flux was found to span 0 to 410 cells/s across all mice, we also examined how similar measurements were for the same capillaries over postnatal weeks 5 to 18. Many high-flux capillaries tended to maintain high flux over weeks (e.g., mouse 04, capillary 07 in Fig. 5C and mouse 01, capillary 05 in Fig. 5D). Many low-flux capillaries tended to stay low over weeks (e.g., mouse 04, capillary 05 Fig. 5C and mouse 01, capillary 02 in Fig. 5D). Still other capillaries had different flux week to week (e.g., mouse 04, capillary 09 Fig. 5C and mouse 01, capillary 06 Fig. 5D)

The average standard deviation from each hyperglycemic capillary tracked over time was 24.34 cells/s. The average standard deviation for each euglycemic capillary was 29.75 cells/s (Fig. 5E). Both hyperglycemic and euglycemic mice showed a similar distribution (kurtosis, 1.97 [hyperglycemic mice] and 1.56 [euglycemic mice]). Generally, lower flux values had a higher overall standard deviation relative to the mean flux. As a fraction of the mean, the capillary variability from week to week was 27% for hyperglycemic mice and 32% for euglycemic mice (Fig. 5F).

### Capillary Blood Cell Flux Is Not Correlated With Elevated Blood Glucose

**Figure 6.**
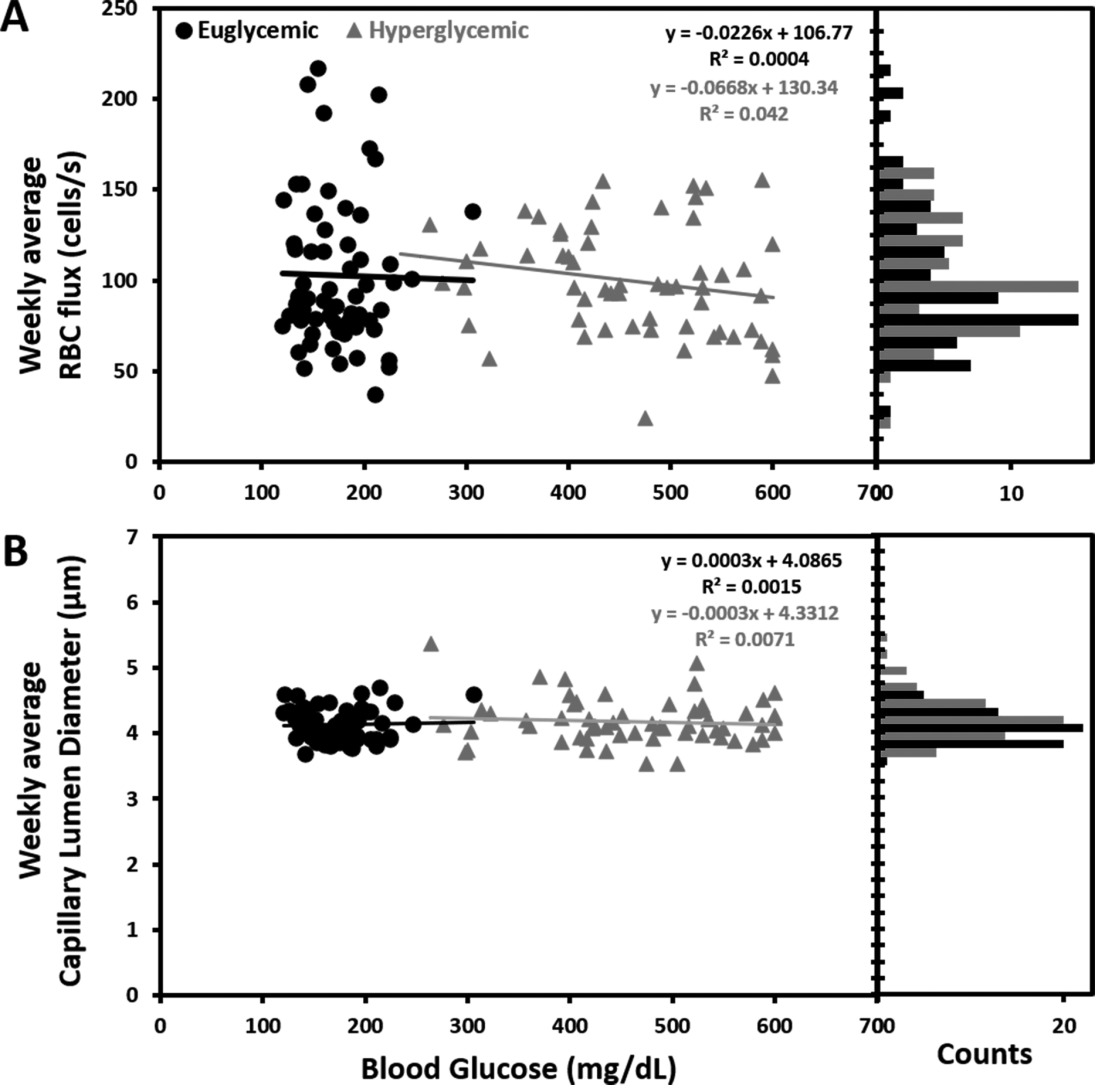
Effect of increased blood glucose on flux and lumen diameter. (**A**) Average weekly RBC flux for euglycemic and hyperglycemic mice as a function of blood glucose. Accompanying bar graph displays flux distribution (12.5 cells/s bins). (**B**) Average weekly lumen diameter measurement for euglycemic and hyperglycemic mice as a function of blood glucose. Accompanying bar graph displays lumen diameter distribution (0.25 µm bins).

We measured the correlation between RBC flux as a function of systemic blood glucose. Independently, the euglycemic and hyperglycemic populations showed a weak correlation between blood cell flux and blood glucose (R^2^ = +0.04 for hyperglycemic mice, slope = −0.02 cells/s per mg/dL and R^2^ = 0.004, slope = −0.07 cells/s per mg/dL for euglycemic mice) (Fig. 6A). When correlating all data from 18 mice, we found a comparably weak association, R^2^ = +0.008 and weak slope association of −0.02 cells/s per mg/dL glucose elevation. The unit (cells/s per mg/dL) describes the expected rate of flux increase for every mg/dL increase in blood glucose, assuming a linear regression. The weekly average RBC flux for all nine euglycemic and nine hyperglycemic mice as a function of their blood glucose are plotted in Supplementary Figure S6.

### Capillary Lumen Diameter Is Not Correlated With Elevated Blood Glucose

Similarly as flux, we measured the correlation between capillary lumen diameter and systemic blood glucose. For both euglycemic and hyperglycemic mice, there was weak correlation (R^2^ = 0.0071, slope = −0.0003 µm per mg/dL for hyperglycemic mice and R^2^ = 0.0015, slope = +0.0003 µm per mg/dL for euglycemic mice) (Fig. 6B), comparable with the correlation for all 18 mice (R^2^ = 0.0025) with the slope of 9^–5^ µm per mg/dL blood glucose elevation. Weekly average capillary lumen diameter for all nine euglycemic and nine hyperglycemic mice as a function of their blood glucose are plotted in Supplementary Figure S7.

### Capillary Stalls Are Rare in Hyperglycemic and Euglycemic Mice

**Figure 7.**
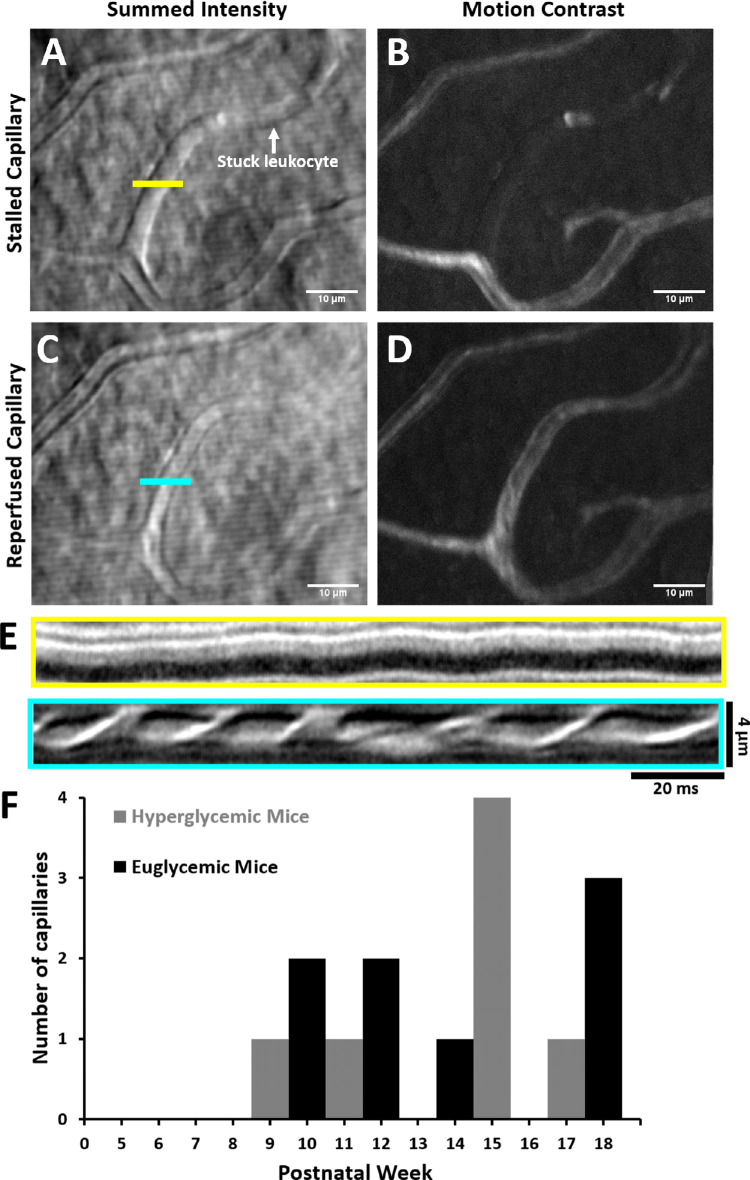
Capillary stalls in vivo. Summed intensity (**A**) and motion contrast (**B**) images of blocked capillary captured with AOSLO. Horizontal line segment denotes location for fast scanner placement. The *white arrow* indicates location of a stationary blood cell. Summed intensity (**C**) and motion contrast (**D**) images of the same capillary later in the same imaging session. (**E**) Space–time line scan images corresponding to blocked (**A**) and perfused (**B**) conditions. (**F**) Capillary stalls quantified from postnatal weeks 5 to 18.

Phase contrast AOSLO enabled the visualization of capillary branches that were perfused or not (Figs. 7A–D). For example, the transient blockage of flow led to the disappearance of the vessel from the motion contrast perfusion map (Figs. 7B, D), yet the capillary was still visible with phase contrast (Figs. 7A, C). When a stall occurs in a capillary, a single stuck cell halts flow in an entire branch (Fig. 7A). Of the 589 capillary segments imaged in euglycemic mice, we observed nine stalls (1.52%, Fig. 7E). Only two of nine stalls were seen to repeat in the same capillary. For hypergycemic mice, there were 7 stalls from 440 capillary segments (1.59%). Only two of seven stalls were seen to repeat in the same capillary. The durations of the stalls were generally seconds to minutes. Both populations showed few to no stalls in young adults. There was a slight increase in the number of stalls as both populations aged, but because the rate of occurrence was so low, a larger data set is needed to examine the statistical significance of this slightly elevated stall rate.

### Precision of RBC Flux Measurements Shows Good Agreement Between Graders

**Figure 8.**
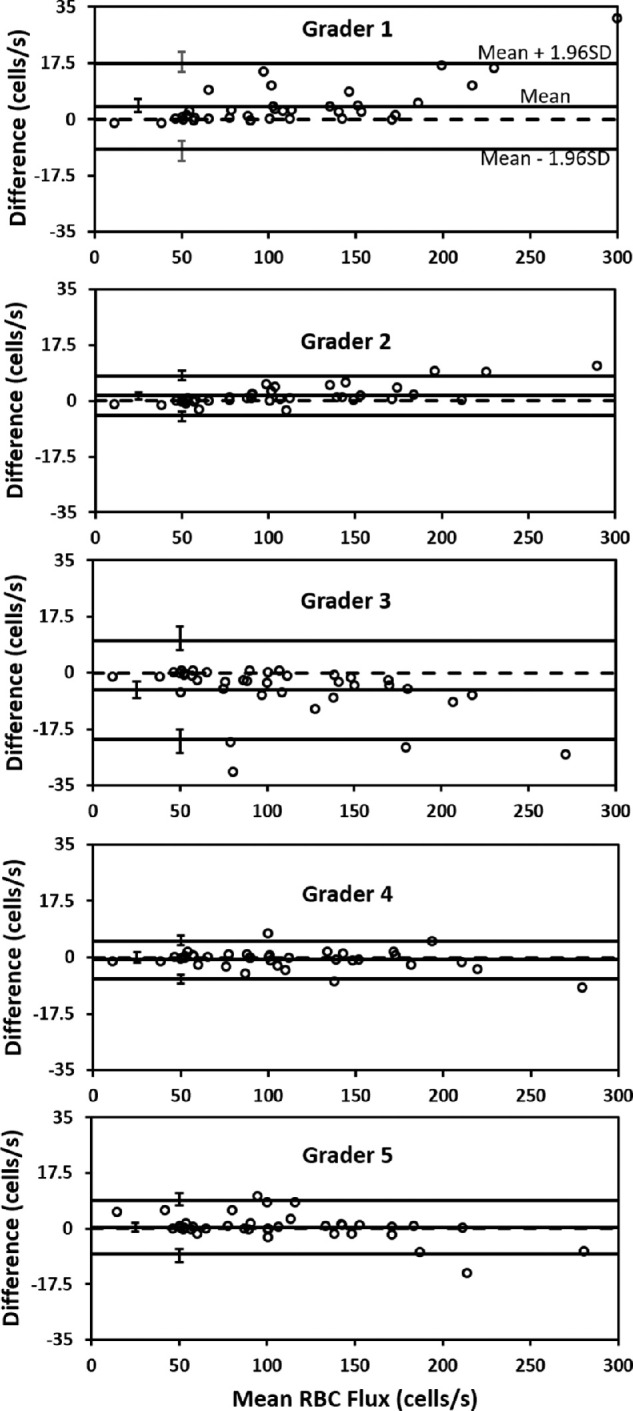
Bland–Altman analysis for manual flux counts. Bland–Altman analysis for five human graders. *Circles* mark manually determined flux values (each grader analyzed 38, 1-second line scans). *Solid horizontal lines* represent the grader mean, ± 1.96 standard deviations (limits of agreement), *dashed line* represents the collective mean for all five graders. Error bars represent 95% confidence intervals for mean and limits of agreement.

Single grader values were plotted against the user average, which was treated as ground truth for Bland–Altman analysis. The highest over counter deviated from the average by 4 cells/s and the lowest undercounter was −5.3 cells/s, indicating that among the five graders, manual flux measurements were precise and repeatable. Ninety-five percent of the flux data for the entire group lied within ±9.8 cells/s difference interval (±1.96 standard deviation); this finding suggests that the repeated manual flux measurements can be reproduced across graders with high precision (Fig. 8).

### Cardiac Pulsatility Is Observed in Capillaries and Adds Variability to RBC Flux Measures

**Figure 9.**
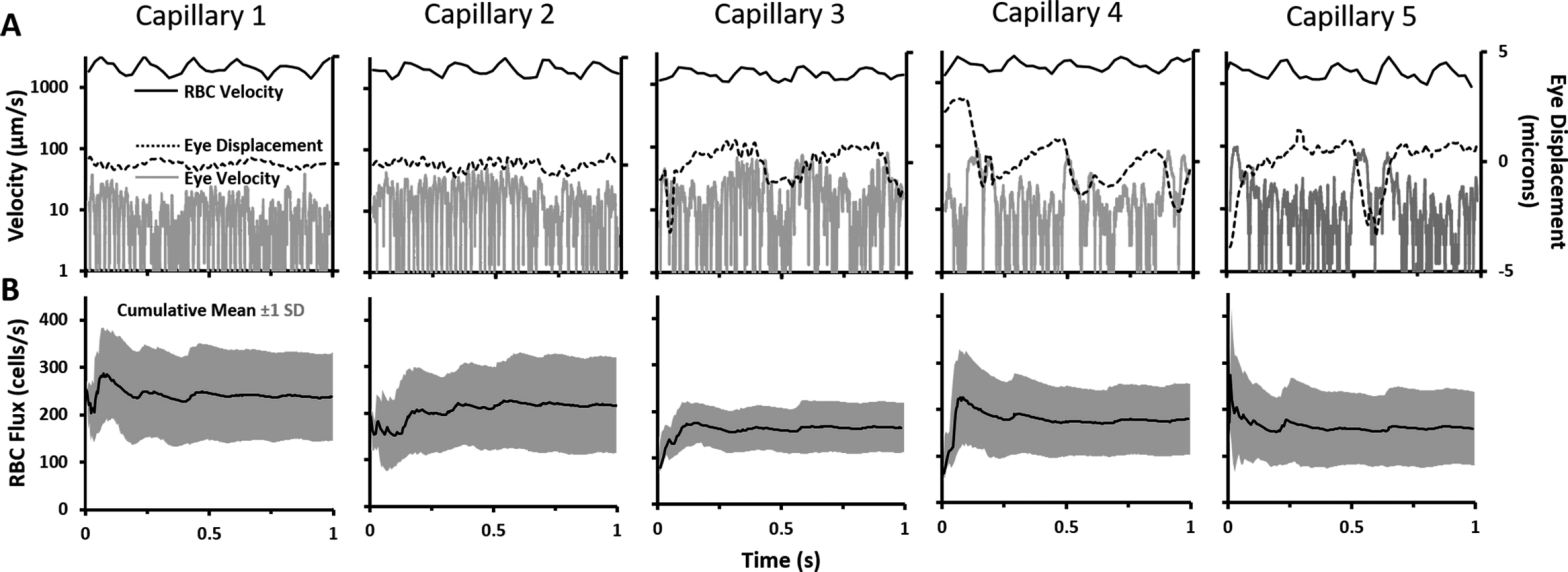
Effects of cardiac pulsatility and eye movement on blood cell flux. (**A**) One-second traces for RBC velocity (*black line*), eye velocity (*gray line*), and eye displacement (*dotted gray line*) for five different capillaries. For each capillary, average eye displacement was set to zero. (**B**) Cumulative mean (*black line*) and standard deviation (*gray shading*) of instantaneous flux, calculated for each corresponding capillary.

Recently, we reported the variability of RBC flux in capillaries is driven partially by the cardiac cycle.^33^ Therefore, we recorded and measured flux over one second of data which comprises multiple cardiac cycles. We find that there are approximately four to five cardiac cycles per second, consistent with the heart rate of the anesthetized mouse^34^ (Figure 9^10^ A, black traces). We find that capillary pulsatility is not an artifact of retinal eye motion. In five capillaries, chosen for their excellent quality and heterogeneous RBC flux (ranged from 126 to 201 cells/s), we quantified the frequency and velocity of RBCs (Fig. 9A, black traces). We also measured the retinal displacement that could, in theory, impart a pulsatile artifact in the data (Fig. 9A, dashed and gray traces, respectively). The global retina motion induced by cardiac cycle, respiratory rate, and other events that move the eye were approximately two orders of magnitude less than the corresponding speed of blood cells. Moreover, the retinal motion was out-of-phase and at a different frequency in all five capillaries analyzed. Combined, this evidence indicates that the RBC pulsatility is not an artifact, but constitutes a physiological behavior of blood cells flowing through capillaries, driven by the cardiac cycle.^33^

**Figure 10.**
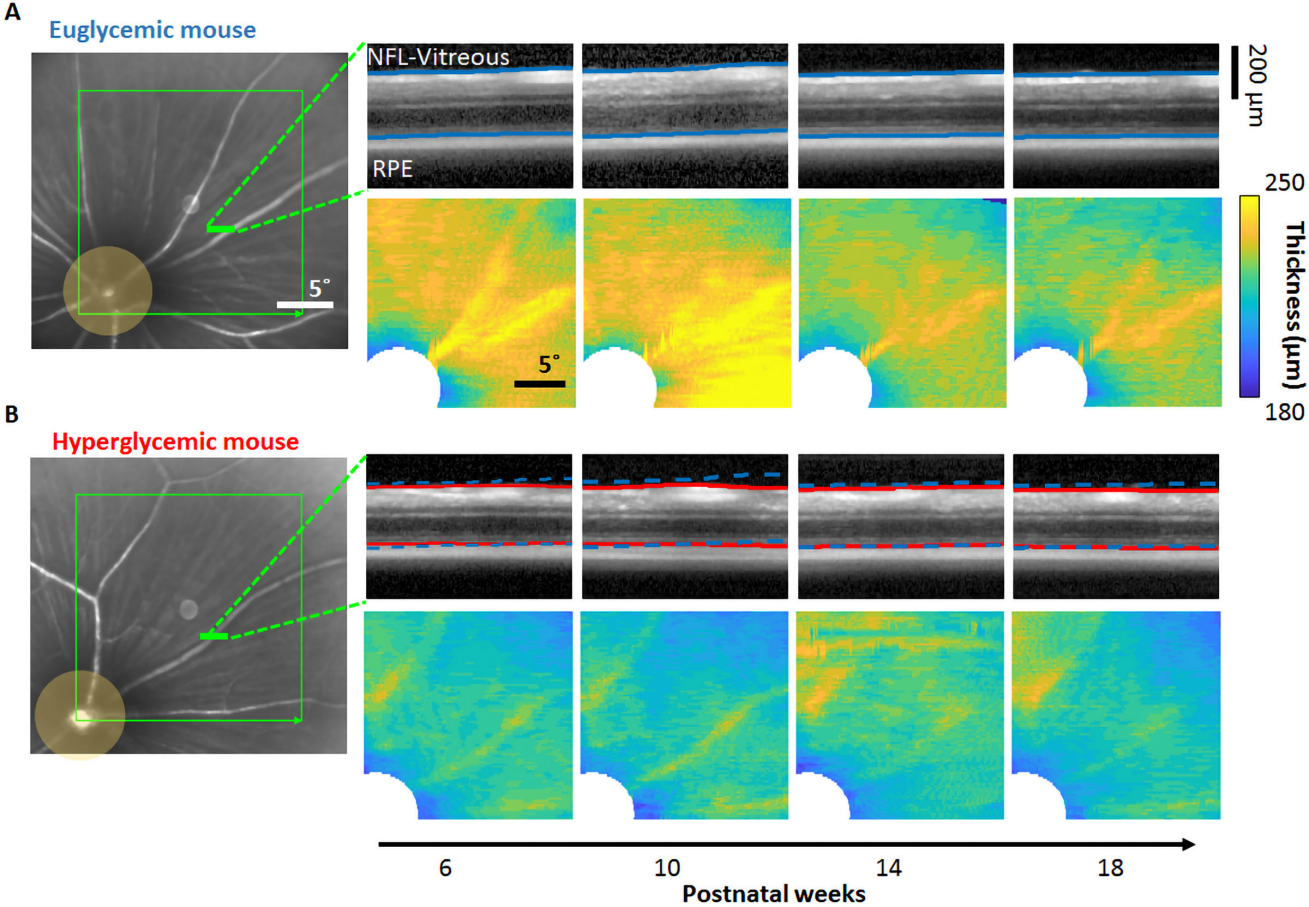
Longitudinal retinal thickness. Longitudinal measurement of retinal thickness in euglycemic (**A**) and hyperglycemic (**B**) mice based on OCT imaging. (*Left*) OCT cubes are acquired at the superior–temporal quadrant of the retina with 20° × 20° FOV (*green box*). (*Right* *top*) Representative OCT B-scan cross-sections (derived from *green line* at left). *Blue and red lines* indicate the NFL–vitreous and RPE layer positions. *Blue dashed lines* in (**B**) indicate the corresponding layer positions of the euglycemic mouse in (**A**). (*Right bottom*) En face total retinal thickness maps sampled from weeks 6 to 18.

### Averaging RBC Flux Over Multiple Cycles Reduces Temporal Sampling Bias

In this study, we did not phase lock data acquisition to the cardiac cycle. Instead, a simple way to mitigate temporal sampling aliasing is to integrate RBC flux measures over a 1-second sampling window which contains four to five cardiac cycles. We show the integrative effects in Figure 9. The cumulative mean RBC flux showed an initial high variability from phase of the cardiac cycle, but was then attenuated after multiple cardiac cycles (Fig. 9B).

### Hyperglycemic Mice Have a Thinner Retina than Euglycemic Littermates From Postnatal Weeks 4 to 20

**Figure 11.**
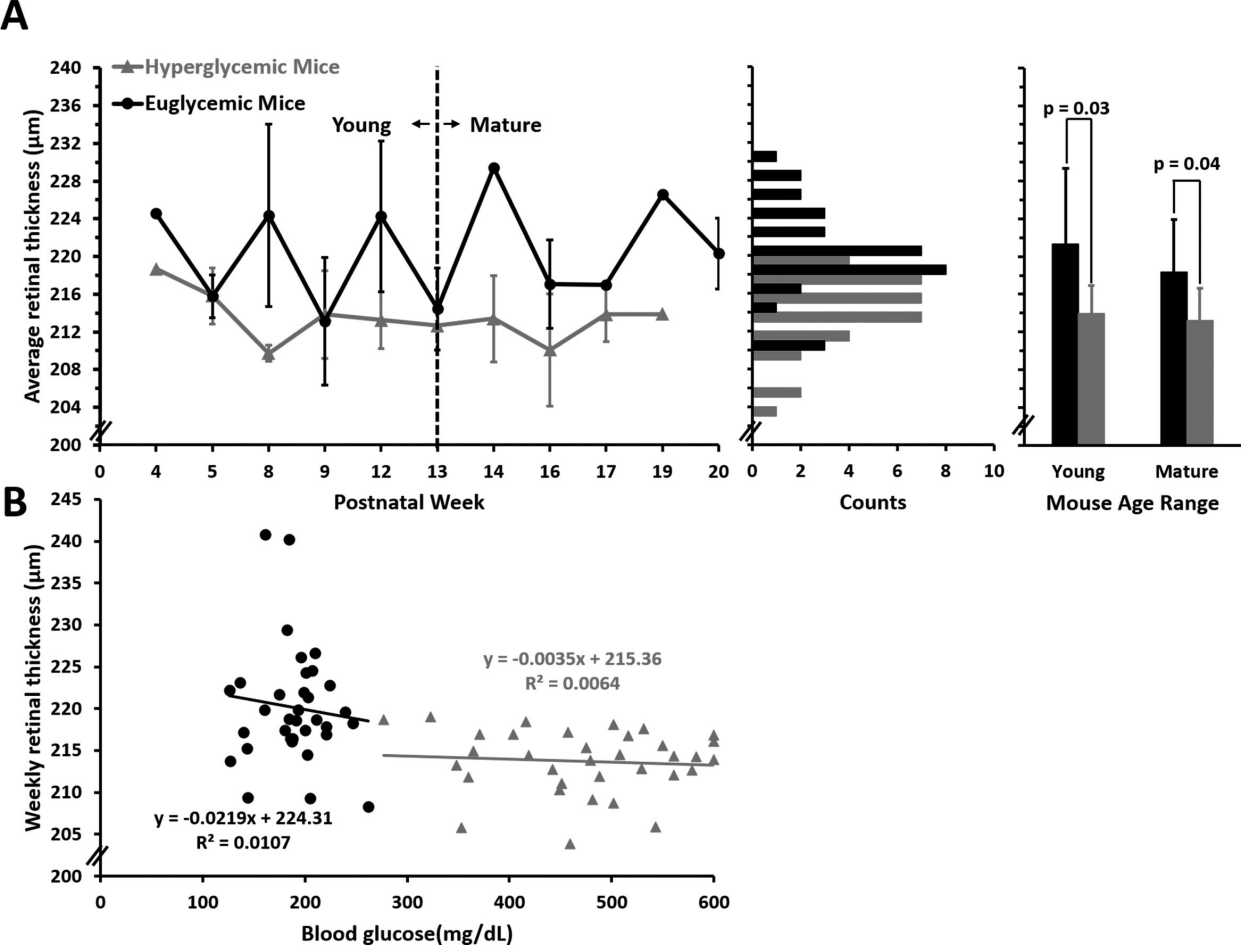
Measurements of OCT thickness across weeks. (**A**) Retinal thickness measured in euglycemic (*n* = 9 mice, *black*) and hyperglycemic mice (*n* = 9 mice, *gray*) plotted from postnatal weeks 4 to 20 (*left*, mean ± SD). Bar graphs (*middle*) display distribution of euglycemic and hyperglycemic retinal thickness from nine hyperglycemic and nine euglycemic mice (bins = 2 µm). Histograms (*right*) display average retinal thickness for young (<13 weeks) or mature (≥13 weeks) mice (mean ± 1 SD). (**B**) Retinal thickness for euglycemic and hyperglycemic mice as a function of blood glucose.

Hyperglycemic mice had a thinner retina as compared with euglycemic littermates between postnatal weeks 4 and 20 (213.7 ± 3.79 µm vs. 221.06 ± 5.79 µm; *p* < 0.001) (Fig. 11A, left). The population of retinal thickness measurements was normally distributed for both euglycemic and hyperglycemic group (Fig. 11A, middle).

This trend was true for both the young and mature time points (Fig. 11A, right). Hyperglycemic retinas were thinner than their euglycemic littermates (213.98 ± 2.76 µm vs. 222.23 ± 5.56 µm; *p* = 0.005) between postnatal week 4 and 12 and between postnatal weeks 13 and 20 (213.23 ± 3.17 µm vs. 219.51 ± 4.66 µm; *p* = 0.011). Although there were differences between hyperglycemic state, we did not observe a statistically significant change in retinal thickness with age in either group (*p* = 0.58 hyperglycemic; *p* = 0.96 euglycemic)

### Retinal Thickness Does Not Change With Elevated Blood Glucose

We did not observe a progressive change in retinal thickness with respect to age and blood glucose with hyperglycemic mice (R^2^ = 0.006; slope = −0.004 µm per mg/dL) nor did we observe a progressive phenotype with euglycemic mice (R^2^ = 0.05; slope = −0.05 µm per mg/dL). When pooling all measurements across all mice (34 OCT cubes each for hyperglycemic and euglycemic mice), a weak correlation was measured between retinal thickness change with increasing blood glucose (R^2^ = 0.23; slope = −0.02 µm per mg/dL) (Fig. 11B).

## Discussion

Since it was discovered that microvascular structure is compromised in diabetic eye disease,^12^^,^^13^ there has remained a standing question of whether microvascular perfusion is impacted before observed anatomic changes. Early changes including microvascular bleeds, microaneurysms, and vascular remodeling have been characterized with histology in the diabetic condition and confirmed with high-resolution in vivo study.^18^^,^^24^^,^^58^ Yet it remains unknown whether blood cell flux is altered in retinal capillaries before even the very first anatomic changes are observed. To address this question, we deployed micron-level resolution adaptive optics imaging that enables the most sensitive measures of blood flow to date, providing measures of RBC flux within the capillaries of the retina without the use of fluorescent dye. In this study, we tracked the same capillaries in hyperglycemic and euglycemic littermates during the rising phase of blood glucose in the Ins2^Akita^ model (postnatal weeks 5 to 18). During this time, mice experience dramatically elevated blood glucose, but only mild behavioral and systemic changes indicated by behavioral monitoring and weight loss (Fig. 1). We find that capillaries do not have altered RBC flux within the central retina despite a two to three fold increase in blood glucose. Therefore, we arrive at the conclusion that elevated blood glucose for weeks to months alone does not impact the perfusion of the retinal capillaries we tracked. We also hypothesized that there could be a subset of capillaries with higher than normal flux that could compensate for capillary pathology (flux reductions and capillary dropout). Such a finding would manifest as a bimodal distribution of RBC flux in hyperglycemic mouse capillaries. Instead, we find that both euglycemic and hyperglycemic capillaries exhibit unimodal gaussian distributions with little observed differences in population spread, indicating there is one dominant population inconsistent with the above hypothesis.

Mice that were tracked in this study were under anesthesia, which permitted stable recording for hours at a time. It should be considered that the absolute flux and velocity measurements reported here could be influenced by anesthesia despite using exceptionally low levels of isoflurane (0.5%–1.0%). Although it is possible that absolute flux numbers could be altered by anesthesia, both euglycemic and hyperglycemic mice were under the same anesthesia protocol and, therefore, it is unlikely that the lack of difference between the populations were attributed to anesthesia. Additionally, we did not observe overt microscopic changes in capillary structure during the early elevated weeks of hyperglycemia (Supplementary Fig. S8). Consistent with other in vivo reports,^59^ we also find that retinae of hyperglycemic mice approximately 7 µm thinner than their euglycemic littermates. This value is small, representing the width of a single cell soma in the retina, yet we find the populations were statistically different. Further studies will need to investigate whether this change in thickness happens in development or represents a real difference in the consequence of hyperglycemia in the adult. Combined, these findings are notable as a number of studies have found a substantial reduction in contrast sensitivity^60^ neural cell loss^39^^,^^61^ and retinal thinning^39^^,^^59^ within the postnatal 17 to 25 week range. Such neural and behavioral findings immediately follow the 5 to 18 week epoch studied here. Although diabetic eye disease is multifaceted, our data give further evidence that the earliest changes in neural diabetic disease may arise from the sequelae of glucose-related neuropathy rather than altered capillary blood flow with a secondary neuroglial phenotype.^3^^,^^62^ We discuss the nuances of our central finding that blood cell flux is not altered despite weeks of elevated blood glucose. We further discuss a number of technical advances that enabled in this study.

## Scientific Advances

### Ins2^Akita^ as a Model of Human Diabetes

**Figure 12.**
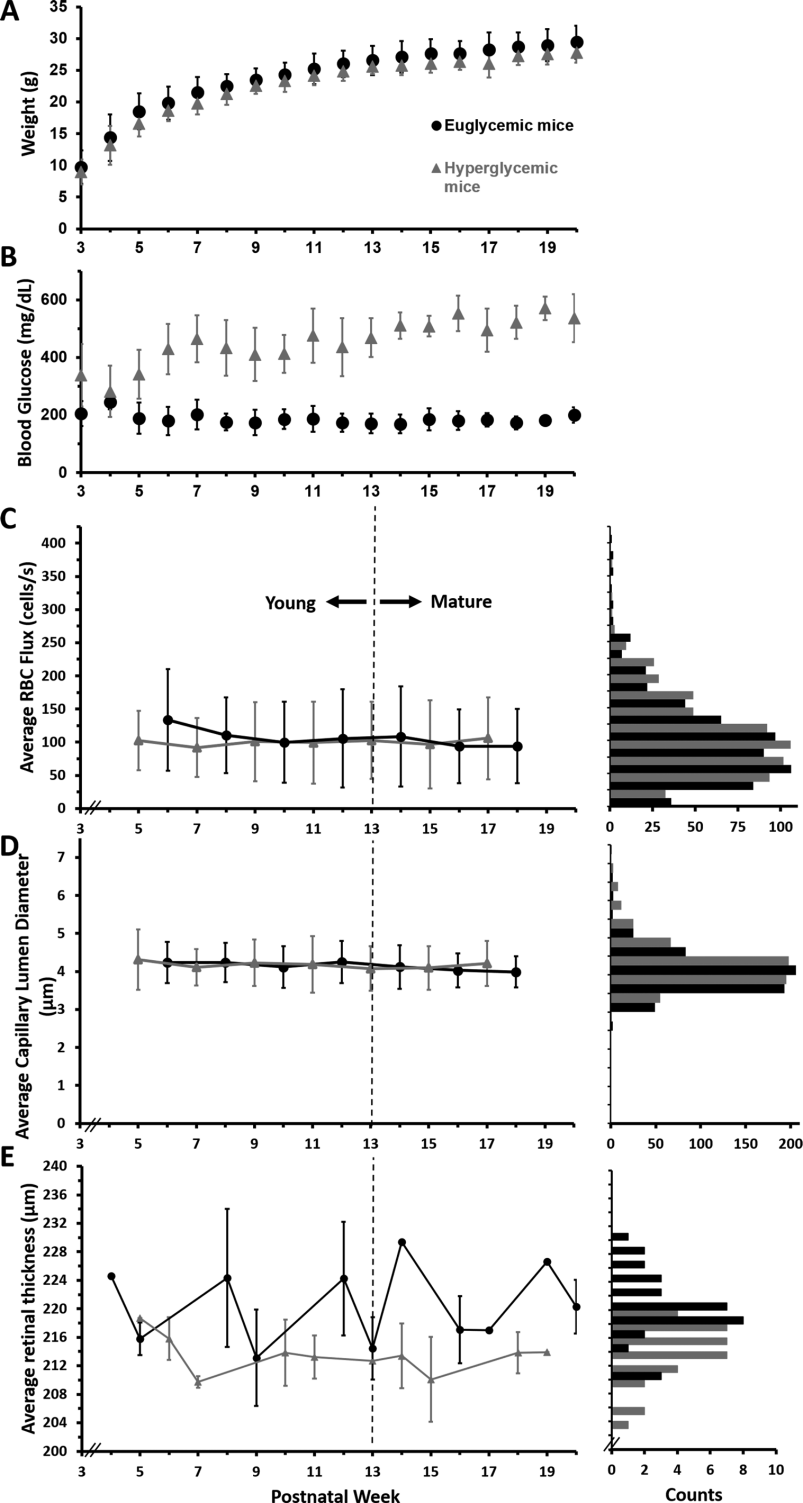
Structural and functional changes in retinal microvasculature, thickness and systemic phenotype in nine hyperglycemic and nine euglycemic mice. Systemic weight (**A**) and blood glucose (**B**) measurements from postnatal weeks 3 to 20. Average weekly RBC flux (**C**, *left*) and capillary lumen diameter (**D**, *left*) measured biweekly between postnatal weeks 5 to 18. The histograms on right display distribution of euglycemic and hyperglycemic flux (bins = 25 cells/s for **C**) and capillary diameter bins = 0.5 µm for **D**); hyperglycemic data was normalized to total euglycemic counts. Average retinal thickness measurements (**E**) measured every 4 weeks from postnatal week 4 to 20. The histograms on right display distribution of euglycemic and hyperglycemic retinal thickness bins = 2 µm.

The Ins2^Akita^ mouse model was chosen to study type 1 diabetes in this study, which has been a popular model for diabetic disease and has been found to display an ocular phenotype.^39^ These mice show an early elevation of blood glucose, starting as early as postnatal week 3. However, this early onset and sustained presence of hyperglycemia does not immediately manifest into changes in the retinal microvasculature commonly associated with DR, as reflected in our findings (Fig. 12). Like any model, it is imperfect and does not capture all aspects of human diabetic disease. Mice do not recapitulate the proliferative stages of late-stage human DR.^3^ Nevertheless, it is an elegant model because the comorbidities, such as hyperlipidemia and hypertension, that often accompany human diabetes do not confound the study of elevated blood glucose alone. Therefore, we are able to study the direct impact of the hyperglycemic condition.

There are a number of other mouse models of type 1 diabetes. Ins2^Akimba^ mice are a transgenic strain obtained from crossing the Akita mouse with the Kimba mouse, which have a transgenic overexpression of human vascular endothelial growth factor. Another is found in the commonly used streptozotocin mouse model, which is imparted by pancreatic toxicity by way of injection. Although the Ins2^Akimba^ model has shown an exacerbated vascular phenotype of DR, it is multifactorial in its deficit, which has the potential to cloud the impact of blood sugar elevation alone, so we considered it important to characterize the vascular properties of its parent Ins2^Akita^ strain to gain a better understanding of how DR develops without altered VEGF expression. We also argue that, because the Ins2^Akita^ mouse model is transgenic, it does not have to factor in potential side effects of streptozotocin-induced hyperglycemia and has the advantage of developing a diabetic phenotype more progressively and consistently without confounds of drug titration, delivery artifact and off-target impacts of the antineoplastic and antibiotic agent.

In humans, there are two notable stages of DR: (1) an early nonproliferative stage characterized by a lack of new blood vessel formation, weakening of vascular lumen and microaneurysms resulting in leakage into the retinal tissue and (2) a proliferative late stage marked by pathological angiogenesis mediated by hypoxia inducible factor and VEGF. Our study design aimed to model the early phase of DR before vascular remodeling or proliferative stages related to hyperglycemia. AOSLO allows us to study the retinal microvessels, which have been poorly studied in vivo, likely attributed to the lack of microscopic resolution of current conventional ophthalmic devices.

### Microscopic In Vivo Histology Reveals no Lumen Diameter Changes in Capillaries

The custom AOSLO system used in our study has less than 1 micron lateral resolution,^63^ granting us the ability to study capillaries in vivo. The advantage of capillary measures in the living eye is that histological artifacts such as fixation tissue shrinkage and vascular collapse are no longer confounding. Combined with phase contrast imaging,^46^^,^^64^^–^^66^ measures of lumen diameter are now possible without the injection of fluorescent dye.^33^ Using this approach, we did not find any evidence of lumen diameter changes in the hyperglycemic or euglycemic populations. Lumen diameters were stable from week to week and averaged 4.1 microns in diameter in the population we studied. This small diameter is notably less than the diameter of an undeformed blood cell requiring the cells the squeeze through this network. Although the definition of capillary is often debated in scientific circles, we emphasize here that all of these vessels were less than 7 microns in diameter and facilitate single file flow regardless of their protein or cellular composition. As such, we did not see evidence of bottlenecks or a decrease in diameter that would have imparted greater resistance on the vascular network. Similarly, we did not see a biphasic diversion of the population whereby some capillaries dilate and others restrict. This finding is consistent with our measures of blood cell flux, which remained largely stable over the first 3 months of diabetic onset despite exhibiting a two to three fold increase in blood glucose.

### AOSLO Reveals No Evidence of Vascular Remodeling in the First 3 Months of Hyperglycemia

Although preclinical features that evaded conventional detection have been observed in human study with adaptive optics,^18^^,^^24^^,^^56^ we did not find evidence of vascular remodeling in the first 3 months of hyperglycemia in mice. There were no signs of hairpin turns, microaneurysms, microvascular bleeds, or ghost capillaries in the areas we monitored. This finding raises questions about how well the lifetime and duration of hyperglycemia allometrically and chronologically scale to the mouse model.^51^ Laboratory mice, with an average life expectancy of 2 to 3 years, are never going to encounter the decades of hyperglycemic insult that diabetic humans experience. Nevertheless, we modeled and imaged the age of mice that best correlates with mature adults^51^ and if scaled in this (imperfect) way, the time points of our mice represent the human 20s, a range where preproliferative changes are seen clinically.^6^ We, therefore, conclude here that epochs of approximately 3 months of dramatically elevated hyperglycemia do not directly perturb capillary anatomy, remodeling or the flux of RBCs within the retina of Ins2^Akita^ mice. We expect further AOSLO investigation will target and investigate this epoch in diabetic humans as a potential early functional point of interest in vascular perfusion before the proliferative phase of disease takes effect.

### New Capabilities in Microscopic In Vivo Imaging Reveal No Early Changes in Capillary RBC Flux

Most studies to date have used ex vivo histology to quantify the structural defects associated with hyperglycemia.^12^^,^^13^^,^^39^ However, these quantified structural changes provide only a single snapshot of the vascular pathology, which cannot encapsulate the interplay and progression of vascular structural and functional changes as it would occur in the living eye. In vivo studies use techniques like intravital microscopy, fluorescein angiography and OCT-A to map microvascular structure and measure lumen diameter and blood flow.^40^^–^^42^ Vascular changes are not observed until at least 6 months of age for Ins2^Akita^ mice, after which a significant decrease in the microvasculature density and blood flow velocity are observed. Our findings constitute a comprehensive longitudinal measure of vascular characteristics that is in agreement with existing literature regarding early epochs of hyperglycemia.

Building on the work of Guevara Torres et al.,^33^ this study represents the first of its kind to evaluate single cell blood flux as a potential functional change in the retina as a consequence of hyperglycemia. Of all the vascular components, it is the capillaries that have been most elusive because of their low endogenous contrast and microscopic size that evades detection in most common ophthalmoscopy. Despite this, capillaries are perhaps the most interesting target in the study of diabetic eye disease (as well as other organs) as it is believed that the anatomic and functional changes at this level are the early underpinnings of progressive and more damaging disease.

Here, we find that in the earliest stages of hyperglycemia, the retinal microvasculature retains its functional integrity despite a dramatic increase in blood glucose. We did observe blood flow stoppages; however, they were rare, on par with the rate of stall occurrence in the cortex.^67^ Moreover, when we observed blood cell stalls, capillaries were reperfused in a span of seconds to minutes for both diabetic and normal mice, suggesting that this is a normal feature of the capillary perfusion in the retina. Collectively, our data suggest that, in this early snapshot of hyperglycemia, there are no substantial changes in capillary perfusion related to hyperglycemia. This finding is notable because many studies have reported vascular changes, albeit at later stages of disease.^39^^,^^40^ This finding would suggest that, in this model, elevation of blood glucose alone takes much longer to change microvascular perfusion. We find it interesting that perfusion is unaltered despite findings from previous studies that contrast sensitivity is reduced to 50% to 60% of normal as early as postnatal weeks 12 to 16.^60^ This outcome would suggest some other neural or behavioral mechanism is attributed to the loss, although one cannot rule out that anterior optic opacification could account for a decrease in contrast sensitivity.

### Ins2^Akita^ Mice Did Not Exhibit Progressive Retinal Thinning

The retinal thickness measured in the healthy euglycemic mice matches a number of previous studies that reported cumulative retinal thickness from the inner limiting membrane to the RPE–photoreceptor complex.^39^^,^^68^^,^^69^ We observed that the hyperglycemic mice have a thinner total retina than the euglycemic mice at all time points, a finding that supports previous reports.^39^^,^^59^ We did not, however, observe a progressive thinning over the postnatal 4- to 20-week interval examined here, indicating that there does not seem to be a change in thickness despite approximately 16 weeks of hyperglycemia. Moreover, the thinning effect seems to be global with no obvious local differences attributed to eccentricities, vessel swelling or shrinking, or regional zones of neural parenchyma deficits between vessels (Supplementary Fig. S8).

A noteworthy finding that bears further study is that, from the onset of this study, the retina of Ins2^Akita^ mice was thinner than littermate controls (Fig. 11). The origin and mechanism of the decreased retinal thickness remains unknown. Previous studies suggested that there is decrease in the inner retinal thickness in hyperglycemic mice, which is possibly contributed by the loss cells in the corresponding layers.^39^ The resolution of the commercial OCT used here was only sufficient to faithfully report total retinal thickness, and we were unable to discern the thickness of individual layers. Layer-specific thickness measures could provide greater insight into which tissue is compromised at or even before the onset of hyperglycemia. Seeing as this study reports no structural or functional vascular defects, we hypothesize a neuro/glial pathology in early diabetes.

## Technical Advances

### Safety and Benefit of Label-free Blood Flux Measurement

By obviating the need for fluorescent agents or tracers, phase contrast imaging using NIR light provided robust quantification of RBC flux. This advance was critical because it enabled longitudinal imaging of the same capillaries over months in the same mice. NIR light required only hundreds of microwatts and was able to provide repeatable measures (Fig. 2) of the same vessels over time based on anatomic landmarks and recorded retinal coordinates. NIR powers were low and within what ANSI considers safe in human subjects (ANSI Z136.1-2014). Beyond safety calculations, we did not see empirical evidence of damage or changes in flux over weeks, indicating that the imaging light was nondestructive to retinal tissue (Figs. 2, 4A). These assets are an essential advance for leaving the neural and vascular system intact without the confounds of dye injection.

### Contrast-free AOSLO Imaging of RBC Flux Compared With Fluorescent Strategies

Because blood cells are microscopic and moving at high speed, many strategies seek to boost the contrast of these cells to aid their detection using fluorescence^70^^,^^71^ or by injecting particles^72^ to aid estimates of vessel flow. This is especially true in the cortex, where two photon imaging has provided the bulk of our understanding of single cell blood flow in the central nervous system.^73^^–^^77^ However, the retina affords a number of advantages including a transparent window through which to view single file blood flow. This thin neural substrate, about 200 µm thick^78^ and containing nonpigmented cellular structure, allows light to easily penetrate it. Additionally, retinal vascular geometry is en face with the imaging plane, unlike the complex axial geometry of the cortex.

Previous studies have used fluorescein isothiocyanate or dextran-conjugated dye injections to boost RBC contrast in the retina by labeling the plasma between cells.^37^^,^^79^^,^^80^ This strategy provides negative blood cell contrast and labels the entire vessel lumen. Still others have succeeded by injecting labeled blood cells^70^^,^^71^ or labeled particles to provide positive contrast to aid retinal velocity detection.^72^

However, our recent advancement uses phase contrast imaging in combination with AOSLO to directly observe the detailed shape, rheology, flow patterns, and contrast of single blood cells using NIR light alone.^33^^,^^46^ Offset aperture detection yields images where erythrocytes display an inverse contrast profile when compared with the capillary walls.^46^ This methodology allows not only detailed counts of cells, but also provides information of the physical deformation of these cells and the complex geometries they possess when compressed within capillaries.^33^ Beyond revealing the shape of the blood cells, here we use the RBC deformation as marker for th*e* patent lumen diameter. Using this strategy provides direct measurement of the inner lumen diameter of capillaries in vivo.

Collectively, a number of imaging milestones have now provided the first measures of single cell blood flow without requiring contrast agents that provide several benefits: (1) off-target effects of dyes do not have the potential to change the rheology, pH, or local hematocrit of blood cells and plasma within vessels.^33^ (2) AO imaging uses NIR light to which the mammalian eye is far less sensitive, minimizing photoreceptor activation and thus any confounds introduced through the neurovascular unit. Mouse visual sensitivity is UV blue shifted relative to human vision, and is thus estimated as more than 12 times less sensitive than humans for red-NIR light attributed in part to lack of long wavelength sensitive cones.^81^^,^^82^ Thus, NIR light is especially advantageous in the retina when compared with the cortex owing to the nature of the sensitivity of the retinal tissue to visible light; the very stimulus it was biologically designed to capture. (3) Counter to popular belief, NIR light provides exceptional contrast of blood cells despite having low chromatic hemoglobin absorption, likely owing to the nature of how contrast is produced using phase contrast approach, discussed further.^46^ (4) Because the phase contrast approach does not require a fluorescent signal, we achieve better stability of recording over long time intervals without dye washout, unstable fluorescence intensity or other physiological compensatory changes to visible imaging light. Finally, (5) because no dyes are injected, the prospect of translational imaging in a clinical setting is attractive to provide such measures in humans.^26^

### NIR Phase Contrast Images of RBCs Provide Exceptional Contrast and Good Count Consistency Across Manual Graders

Although it may seem that the phase contrast approach may provide less contrast compared with fluorescent labeling techniques,^79^^,^^80^ we found strong RBC contrast was provided using the cellular refraction and scatter of NIR light.^33^^,^^46^ Moreover, the detailed topographical shape of single blood cells is revealed, whereas such detail is often missing using fluorescence approaches. One metric that demonstrates the robustness of the collected data is revealed in the Bland–Altman analysis that examines the consistency of counts between graders. On a randomized data set of 38 capillary segments, we found that the bias for each of five graders was near zero (5.3 cells/s was the highest user bias) indicating that, on average, users did not tend to overcount or undercount RBC flux compared with their peers. Differences in counts were small (Fig. 9) and the 1.96 standard deviation threshold in the Bland–Altman calculation showed only 9.8 cells/s difference in counts which is low with respect to the biological variability we observed in capillaries owing to nonperiodic RBC flux from heart rate and other stochastic vascular processes^34^ (Fig. 9). Therefore, there is strong agreement in counts indicating high precision across graders, suggesting the manual flux counts reported in this report are reliable.

### Temporal Requirements to Measure Capillary Flux

The present study imaged capillaries at high speed (15.45 kHz) using the line scan modality combined with adaptive optics.^33^^,^^83^ By comparison, exact counts of blood cells in retinal capillaries have only been possible in a small number of studies.^26^^,^^33^^,^^34^^,^^79^^,^^80^^,^^84^ Notably, we see RBC flux rates often higher than those reported in other studies. Here, RBC flux as high as 410 cells/s was observed in capillaries which sets the Nyquist sampling rate for such analyses to be more than 800 Hz when captured orthogonal to a vessel. This value is important when considering other approaches often use frame or line rates at or below what is required to see the full spectrum of RBC flux rates in capillaries (650 Hz),^80^ or similar modalities for image acquisition (800 FPS).^26^ The importance of Nyquist sampling and recovering undersampled data is discussed in detail by Bedggood and Metha.^85^ Kornfield and Newman^79^ have reported flux values substantially lower (approximately 39–61 cells/s baseline) in the rat. This factor may represent a difference in species, imaging preparation, or the aforementioned Nyquist limits for calculation. For our data, we are confident that 410 cells/s represents the true upper limit of observed capillary flux as our sampling frequency of 15.45 kHz is more than 19 times the temporal Nyquist limit. In other words, it is theoretically possible to image more than 7000 cells/s in single file using this approach. Related to this large temporal bandwidth, it means that adjacent lines in such images may be averaged by approximately 19 times to increase the signal to noise ratio without temporal aliasing. The ability to temporally average adjacent lines enables a higher signal to noise ratio that avoids detector and sources of electronic noise that would degrade low-power NIR light detection and contrast.

### Translation to Human Study

As a forward-looking statement, we expect these advances will open new lines for study of microvascular perfusion in the living retina. This supposition holds its own merit to better understand microvascular disease associate with many retinopathies, but also may provide a glimpse of central nervous system disease, aging, and pharmaceutical response to therapy. Recently, Gu et al*.*^26^ have deployed the use of AOSLO technology to image perifoveal capillaries of the human eye, they reported blood cell metrics from 92% of their spatiotemporal scans, compared with 96.3% for this study. Although we must contend with the optical limits of the human eye that are inferior to the numerical aperture of the mouse for imaging^86^ (human N.A. 0.2, mouse N.A. 0.5), we expect an approximately 2-micron resolution achieved in the human eye with adaptive optics will be an enormous gain over conventional imaging strategies that provide little information of the capillaries, or spacing between adjacent blood cells necessary to conduct this type of analysis.

Although this study represents the first of its kind and a numerically large data set, we recognize that larger studies with more capillaries, more mice, and more time points may shed further light on the nature of the early hyperglycemic changes in the eye. Here, we are tracking 18 mice, and of those, 6 to 14 capillaries among thousands per mouse. It is possible that other capillary segments did reveal changes and our study missed them, however we observed a lack of an obvious capillary phenotype in any of the nine hyperglycemic mice, at any time point. Six to 14 single capillaries tracked are more than any other study that we are aware of to date. Moreover, we performed staggered OCT imaging on the same cohort of mice and we did not observe geographic areas of interest showing swelling, edema, or gross vascular changes using widefield SLO or OCT imaging over the central 35° of visual angle. This finding would indicate that we did not accidentally miss geographically unique areas that are remodeling or changing flow. Instead, our interpretation of these new data is that there is minimal if negligible change in blood cell flux or capillary lumen diameter in the population of retinal capillaries that are all subjected to extremely high levels of blood glucose between postnatal weeks 5 and 18 in the mouse.

## Supplementary Material

Supplement 1

Supplement 2
