# Supplementary material for: In Vivo Capillary Structure and Blood Cell Flux in the Normal and Diabetic Mouse Eye
Source: Invest Ophthalmol Vis Sci. 2022 Feb 9;63(2):18. doi: 10.1167/iovs.63.2.18 (PMC8842443; doi:10.1167/iovs.63.2.18)
Supplement: Supplement 1 [file iovs-63-2-18_s001.pdf]

Supplementary Tables:

	Weekly weight measurements for 9 Euglycemic Mice (grams)								
	Mouse Number								
Postnatal week	01	02	03	04	05	06	07	08	09
3	12.82	6.47	11.63	11.6	7.5	4.4	10.3	10.16	12.62
4	13.74	10.3	18.1	18.2	13.5	6.1	14.49	15.19	18.22
5	19.35	15.9	21.32	22.4	18.89	11.85	19.03	17.78	20.61
6	20.74	18.26	22.79	21.67	20.19	13.25	20.52	19.74	22.3
7	22.4	19.1	24.75	24.56	21.23	15.93	22.34	20.54	22.3
8	24.32	20.45	24.86	25.36	21.15	19.78	23.29	20.82	21.77
9	25.38	22.03	24.32	25.91	22.95	20.01	23.41	21.41	24.94
10	26.58	23.3	25.09	26.3	23.37	20.97	24.21	21.86	26.29
11	26.9	22.1	28.44	28.43	24.24	21.76	25.92	22.04	26.28
12	28.03	25.08	28.18	27.61	25.08	22.03	27.5	22.7	27.42
13	29.32	25.31	29.04	29.16	25.2	23	26.76	22.75	27.49
14	29.82	25.52	30.14	30.28	25.56	23.33	27.4	23.32	28.56
15	29.29	25.99	30.36	30.5	26.49	24.39	27.78	23.65	29.1
16	30.29	26.88	29.3	29.1	26.68	24.83	27.88	23.55	29.04
17	32.1	27.7	30.84	30.65	25.55	23.54	28.69	24.54	29.72
18	31.6	27.52	30.78	30.41	27.54	25.89	28.33	24.41	31.3
19	32.3	29.01	31.38	31.03	28.08	24.7	29.04	24.54	30.95
20	33.84	28.73	31.95	30.92	28.59	25.62	29.2	24.94	31.03
21	35.16	29.36	31.95	30.84	25.05	22.48	29.95	25.31	30.72
22	35.06	29.6	32.53	32.33	28.66	26.28	31.14	25.65	31.83
23	36.47	29.63	32.51	32.71	28.95	26.28	30.72	25.77	33.04
24	37.38	30.03	33.39	33.16	29.01	26.75	31.52	26.5	34.06
25	36.43	27.95	33.17	33.09	30.1	27.22	31.5	26.01	35.32
26	38.85	30.04	33.7	33.31	32.06	28	32.42	26.18	35.37
27	40.18	30.69	34.5	34.56	30.37	27.47	32.21	26.24	34.85
28	40.63	31.29	34.79	34.17	29.58	27.07	32.1	27.07	36.01
29	40.89	32.23	34.14	34.26	30.01	27.32	32.27	26.99	37.48
30	40.98	31.78	34.3	34.54	31.15	28.61	31.62	27.05	37.5
31	41.04	32.13	34.95	34.23	31.55	28.99	32.48	26.94	37.84
32	40.94	32.48	34.18	34.31	31.58	28.78	32.5	27.28	38.06
33	41.58	32.57	35.58	34.49	31.65	28.8	32.57	27.1	37.95
34	40.65	31.95	35.69	34.54	31.02	28.51	32.89	26.82	38.19
35	41.49	33.34	35.14	34.57	31.88	28.9	31.54	27.1	38.72
36	43.55	32.75	34.76	34.21	32.02	27.64	32	27.48	39.19
37	43.64	32.9	35.02	33.89	31.93	28.01	32.86	27.51	39.88
38	42.19	32.58	35.49	34.81	32.31	28.33	32.49	27.29	40.69
39	43.87	33.09	36.17	34.66	32.5	28.5	32.31	27.33	40.9
40	43.89	34.16	36.09	35.01	32.75	28.75	33.05		

Supplementary table 1: Weekly weight measurements (in grams) for 9 euglycemic mice from postnatal week 3-40

Weekly weight measurements for 9 Hyperglycemic Mice (grams0									
Mouse number									
Postnatal Week	01	02	03	04	05	06	07	08	09
3	9.24	9.68	11.69	6.84	5.1	10.1	10.23	7.47	10.28
4	7.65	10.3	17.34	12.3	10.6	16.15	15.43	13.19	15.48
5	12.06	16.33	20.08	17.36	15.84	17.21	17.55	15.44	17.4
6	16.27	20.08	20.43	17.69	17.35	21.51	18.67	17.05	18.67
7	17.46	19.98	23.96	18.48	18.83	21.16	19.43	18.62	20.32
8	18.47	21.08	24.86	20.29	22.5	22.79	21.15	19.33	21.44
9	21.19	23.25	25.3	21.18	22.79	23.42	22.89	21.17	22.17
10	22.48	24.99	25.87	21.77	25.02	24.1	23.17	21.86	20.53
11	22.76	24.4	26.8	22.36	25	25.8	23.68	22.11	24.36
12	24.02	25.29	27.68	23.35	26.05	25.97	24.22	22.35	24.66
13	24.53	25.81	27.14	24.15	27.7	26.29	25.2	23.9	25.5
14	24.56	25.82	28.58	24.39	26.63	26.72	25.65	23.15	25.98
15	25.05	26.79	28.44	24.79	24.8	27.4	25.83	23.99	27.39
16	25.18	27.07	28.6	25.63	27.69	26.96	26.01	23.85	26.32
17	26.07	28.15	28.23	22.11	23.43	28.38	25.92	24.49	27.45
18	26.17	28.22	28.9	27.38	28.68	28.67	26.41	24.21	26.85
19	26.89	28.73	30.55	27.89	27.92	28.42	26.43	24.24	26.66
20	26.72	29.06	30.56	28.03	28.03	29	26.82	24.55	27.43
21	26.61	28.9	30.33	20.68	20.82	28.35	27.78	24.98	27.64
22	27.44	29.64	31.39	28.82	28.9	30.29	28.84	25.85	29.16
23	27.64	30.69	30.79	28.93	29.12	29.73	28.17	26.03	27.73
24	28.46	30.83	30.28	30.34	29.5	30.72	28.5	28.57	28.59
25	24.08	24.61	31.41	30.64	30.44	29.88	28	26.61	27.8
26	28.71	30.29	32.11	28.36	28.24	30.7	28.08	26.65	27.86
27	28.74	30.5	31.48	30.07	29.56	30.07	28.05	25.98	28.65
28	28.55	30.23	32.18	28.51	26.5	30.24	28.86	21.94	20.34
29	29.55	31.68	31.02	28.28	26.56	30.61	28.93	27.09	29.01
30	29.82	32.02	31.9	28.95	27.95	30.3	29.01	27.15	28.87
31	30.15		31.18	20.28	20.27	30	29.18	27.97	28.52
32	28.81		32.83	30.03	28.51	29.94	28.95	27.25	28.01
33	28.85		32.98	29.92	28.23	29.15	28.6	27.01	27.56
34	27.96		33.2	29.9	27.91	29.18	28.92	26.93	26.19
35	29.49		33.18	29.54	28.1	30.05	29	26.47	27.63
36	30.78		32.59	28.83	28.38	29.98	28.96	27.15	27.4
37	30.69		32.54	29.04	28.65	29.4	28.76	26.03	26.49
38	30.22		32.87	29.15	27.74	28.99	26.49	28.39	
39	30.87		31.86	29.29	28.43	28.42	26.3	28.36	
40	29.95		32.04	29.5	29.09	29.15			

Supplementary table 2: Weekly weight measurements (in grams) for 9 hyperglycemic mice from postnatal week 3-40

	Weekly blood glucose measurements for 9 Euglycemic Mice (mg/dL)								
	Mouse Number								
Postnatal week	01	02	03	04	05	06	07	08	09
3	235	284	205	145	233			147	185
4	222	228	273	227	249		288	207	262
5	250	269	128	155	143	127	247	151	174
6	160	148	138	172	154	225	306	176	139
7	160	154	241	211	183	329	214	188	195
8	161	201	175	187	229	202	121	149	184
9	131	133	128	144	262	221	221	175	186
10	176	229	224	120	195	216	196	166	140
11	152	211	194	196	238	133	276	196	149
12	184	203	211	193	135	151	214	126	136
13	246	165	131	141	144	188	200	178	132
14	182	146	191	145	133	169	181	132	170
15	205	144	183	154	156	230	125	230	239
16	227	202	191	160	148	136	205	140	224
17	141	160	214	165	189	221	195	190	172
18	145	142	147	167	185	180	196	166	186
19	210	161	185	159	162	201	192	186	196
20	163	180	239	199	208	255	187	192	174
21	256	181	250	223	124	157	230	181	196
22	164	175	203	174	188	195	189	160	185
23	206	183	211	250	195	159	181	176	217
24	242	179	195	172	211	211	172	197	156
25	179	184	148	161	171	171	223	173	182
26	238	231	191	142	160	160	151	184	165
27	180	198	249	235	169	169	200	189	237
28	220	194	210	170	172	172	237	287	172
29	229	155	212	215	193	193	213	132	171
30	215	160	194	206	159	159	142	171	144
31	164	169	141	176	165	165	160	185	157
32	221	191	253	194	222	222	150	196	153
33	205	146	214	187	165	165	150	144	202
34	204	191	186	155	201	201	187	128	145
35	234	225	202	188	189	189	163	197	198
36	161	219	251	154	143	143	121	208	200
37	169	191	185	122	196	196	185	165	188
38	188	155	142	189	257	257	202	191	200
39	197	186	188	193	172	172	193	193	212
40	162	200	192	185	180	180	205		

Supplementary table 3: Weekly blood glucose measurements (in mg/dL) for 9 euglycemic mice from postnatal week 3-40

	Weekly blood glucose measurements for 9 Hyperglycemic Mice (mg/dL)								
	Mouse Number								
Postnatal Week	01	02	03	04	05	06	07	08	09
3	393	248	559	309			361	292	205
4	447	155	344	300	365	201	206	301	224
5	376	305	392	264	285	436	502	236	277
6	416	442	531	349	535	561	365	323	341
7	340	345	524	449	481	600	497	531	416
8	298	505	440	501	600	508	338	352	354
9	337	495	357	416	504	588	371	303	323
10	360	404	502	416	547	413	348	353	371
11	573	473	600	451	600	475	395	314	400
12	300	588	540	344	541	438	347	340	485
13	439	522	406	422	579	463	424	392	572
14	488	550	457	555	503	479	508	459	600
15	499	524	434	561	543	513	535	491	480
16	410	548	501	532	593	600	600	597	600
17	362	467	419	530	436	600	589	522	522
18	529	516	419	544	563	600	441	502	583
19	542	468	599	600	600	595	583	563	588
20	491	600	572	600	600	558	452	349	600
21	563	591	587	571	600	600	479	370	600
22	551	600	556	600	600	600	514	426	575
23	598	600	600	584	600	600	536	447	600
24	600	600	533	600	600	600	542	403	496
25	371	600	472	600	600	535	541	500	600
26	600	600	456	600	600	560	555	584	600
27	600	600	600	600	600	542	588	490	587
28	600	600	578	600	600	600	585	306	550
29	560	600	600	600	600	600	591	536	574
30	537	600	555	600	600	570	600	573	600
31	600		563	600	600	600	576	600	600
32	600		575	600	600	600	600	600	600
33	600		600	600	600	592	600	600	588
34	600		600	600	600	540	512	598	513
35	600		588	600	600	600	598	532	600
36	600		600	600	600	600	577	600	600
37	600		600	600	600	600	583	600	600
38	600		600	600	600	599	600	600	
39	600		592	600	600	600	600	600	
40	600		600	600	600	600			

Supplementary table 4: Weekly blood glucose measurements (in mg/dL) for 9 hyperglycemic mice from postnatal week 3-40

	Image Magnification (um/1 degree)
week 5	32.05
week 6	32.26
week 7	32.47
week 8	32.68
week 9	32.89
week 10	33.1
week 11	33.31
week 12	33.52
week 13	33.73
week 14	33.94
week 15	34
week 16	34
week 17	34
week 18	34

Supplementary Table 5. Age based magnification scaling for the growing C57BL6/J mouse eye (derived from [49]

	Total measurements	Min Flux	Max Flux	Mean Flux	Min Diameter	Max Diameter	Mean Diameter
Guevara-Torres et al. 2016	99	17	539	159	3.2	6.5	4
Current study: Euglycemic	548	5	410	106	2.8	6.8	4.1
Current study: Hyperglycemic	405	3	357	102	2.9	7	4.1

Supplementary Table 6. Comparison of capillary Flux/diameter metrics with previous study[33]
