# Supplementary material for: In Vivo Capillary Structure and Blood Cell Flux in the Normal and Diabetic Mouse Eye
Source: Invest Ophthalmol Vis Sci. 2022 Feb 9;63(2):18. doi: 10.1167/iovs.63.2.18 (PMC8842443; doi:10.1167/iovs.63.2.18)
Supplement: Supplement 2 [file iovs-63-2-18_s002.pdf]

Supplementary figures

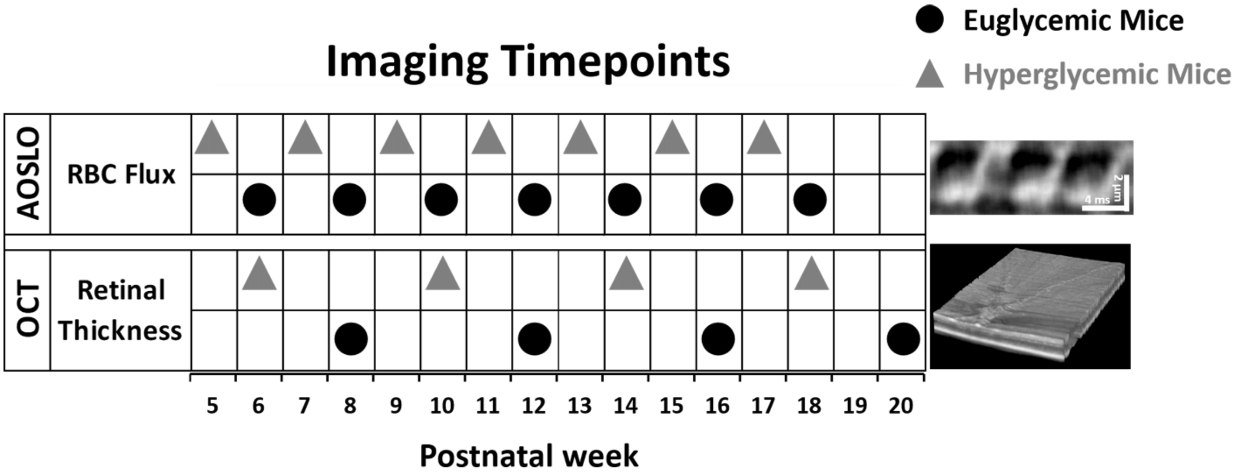

Supplementary figure 1: Timeline of Imaging:

RBC Flux was imaged with an AOSLO. Two populations were imaged in a staggered manner at biweekly intervals. Hyperglycemic mice (grey triangles) were imaged from postnatal week 5-17. Euglycemic mice (black circles) were imaged from postnatal week 6-18. OCT and SLO images were captured every four weeks at a staggered interval. Hyperglycemic mice, postnatal week 6-18 and euglycemic mice ,postnatal week 8-20.

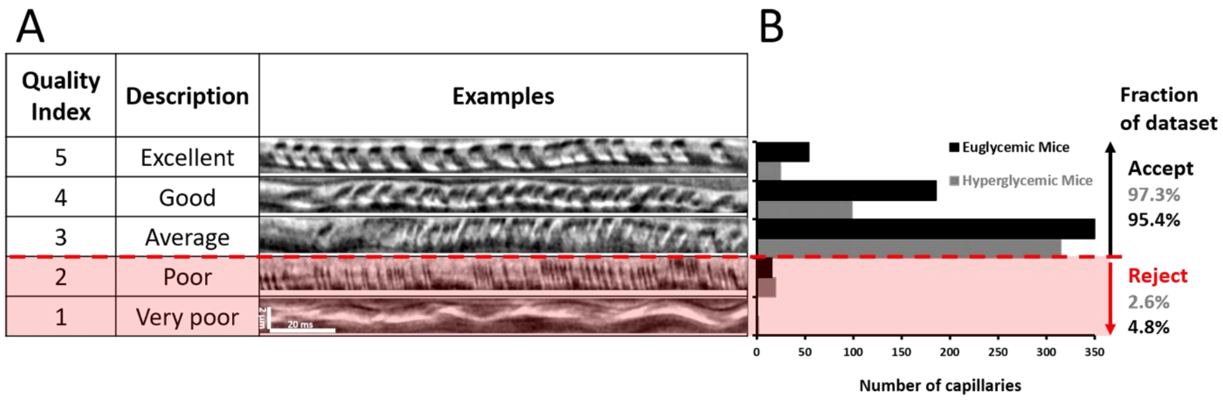

Supplementary figure 2: Subjective Quality index for RBC flux and lumen diameter A) Based on subjective assessment of image contrast and discriminability of individual cells, a human grader assigns a single value to the quality of the imaged data. Representative examples shown

at right. **B)** Histogram of the quality ranking of all data from euglycemic and hyperglycemic mice. Capillaries categorized as “poor” or “very poor” represented 2.6% of total euglycemic capillaries and 4.8% hyperglycemic capillaries. This fraction was not included in RBC flux and diameter analysis (red shaded area, below red dotted line)

A.

Weeks tracked	Hyperglycemic Mice		Euglycemic Mice	
	Capilllaries	% of total	Capilllaries	% of total
1/7 weeks	2	100	0	100
2/7 weeks	3	97.67	0	100
3/7 weeks	7	94.19	0	100
4/7 weeks	11	86.05	4	100
5/7 weeks	28	73.26	17	95.88
6/7 weeks	18	40.7	44	78.35
7/7 weeks	17	19.77	32	32.99
**Total capillaries**	**86**		**97**	

B.

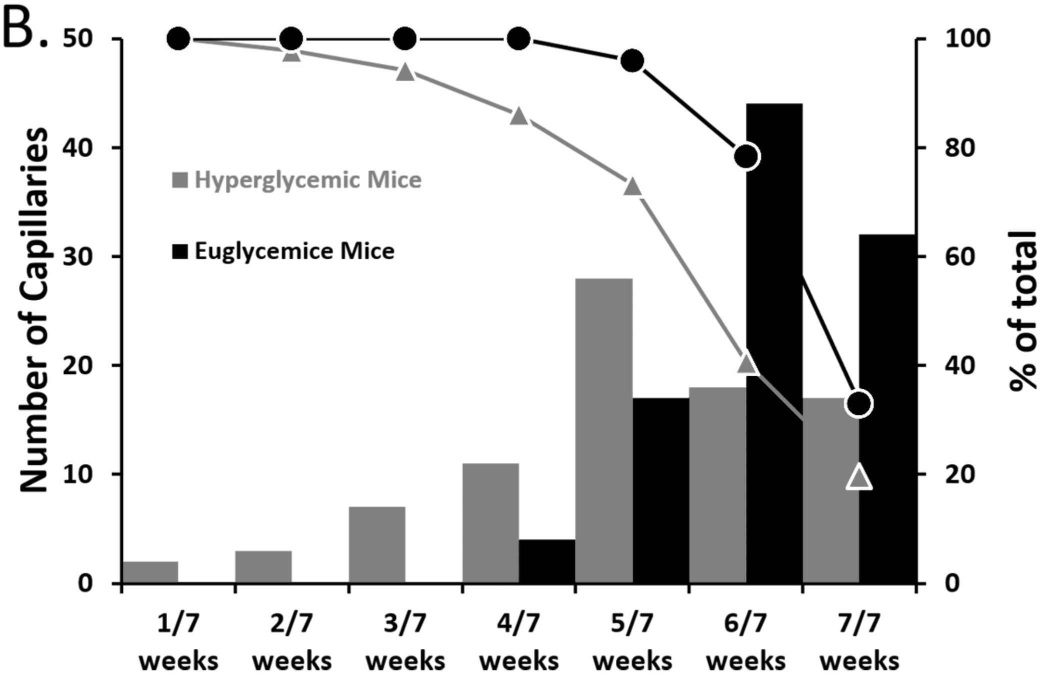

Supplementary figure 3: Longitudinal imaging rate of success for RBC flux A) Number of capillary segments tracked successfully for hyperglycemic and euglycemic mice across 7 imaging time points in postnatal weeks 5-18 along with their cumulative distribution. Total 86 unique capillaries were tracked for hyperglycemic mice, and 97 for euglycemic mice. **B)** Histogram

showing the number of capillary segments tracked successfully for hyperglycemic (gray) and euglycemic (black) mice, along with their cumulative distribution (line plots)

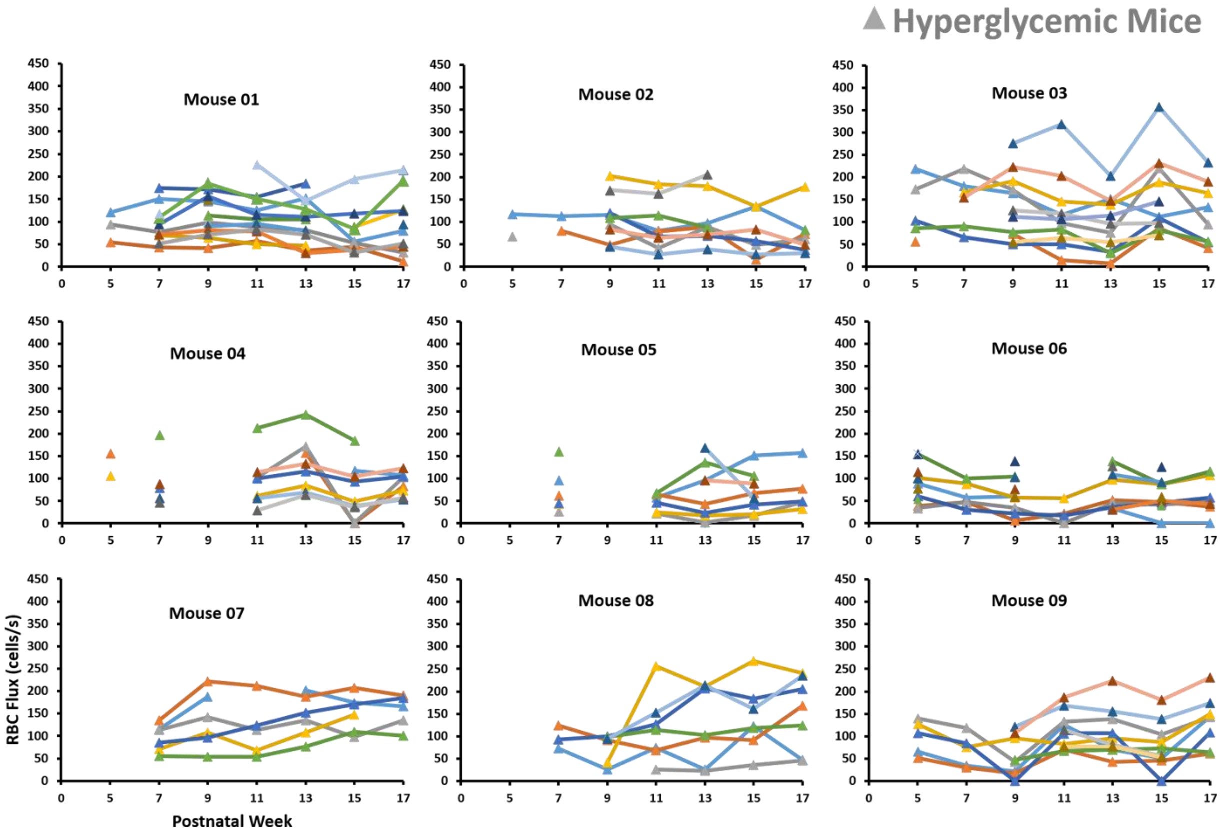

Supplementary figure 4: RBC capillary flux in hyperglycemic mice

RBC flux in capillaries for 9 hyperglycemic mice were longitudinally tracked biweekly across postnatal weeks 5-18. Each line in a plot represents one capillary. Breaks in each line represents missing data due to preparation variables and quality of ocular preparation. 6-14 capillaries were tracked in each mouse.

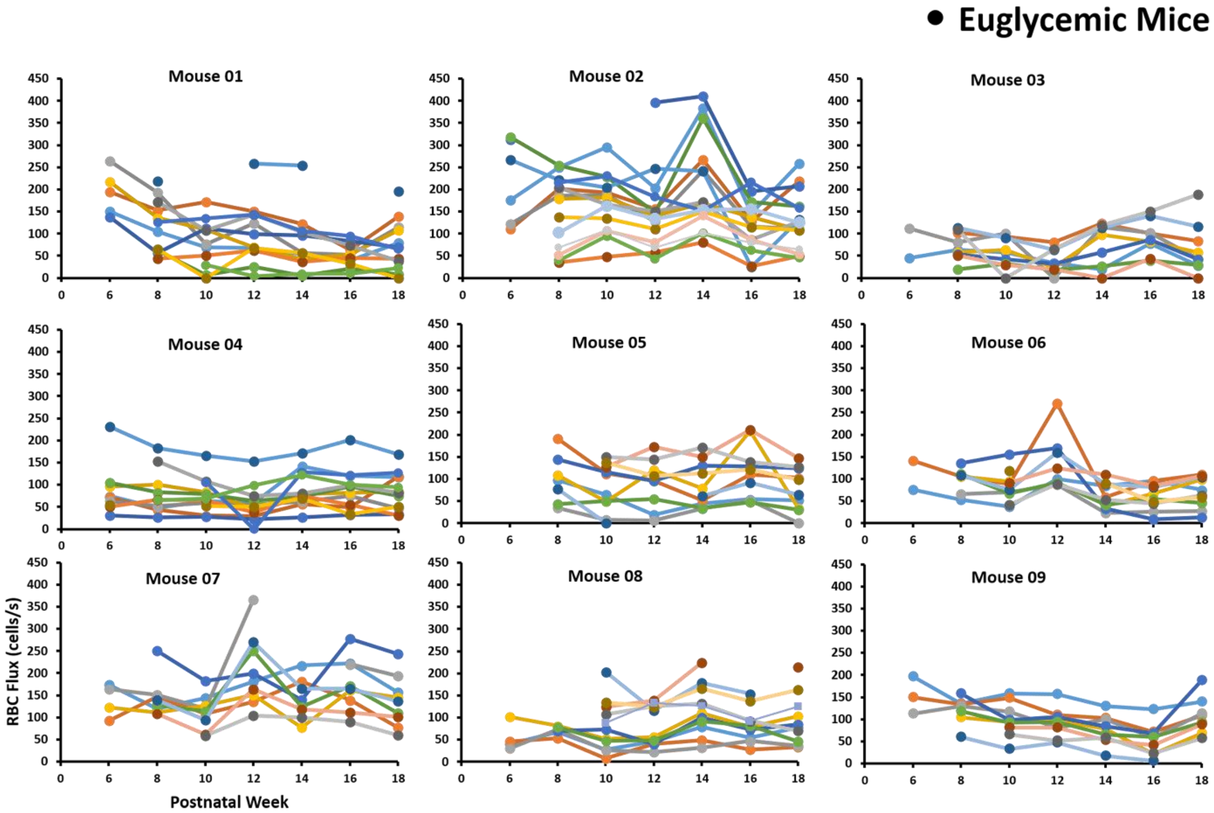

Supplementary figure 5: RBC capillary flux in euglycemic mice

RBC flux in capillaries for 9 euglycemic mice were longitudinally tracked biweekly across postnatal weeks 5-18. Each line in a plot represents one capillary. Breaks in each line represents missing data due to preparation variables and quality of ocular preparation. 6-14 capillaries were tracked in each mouse.

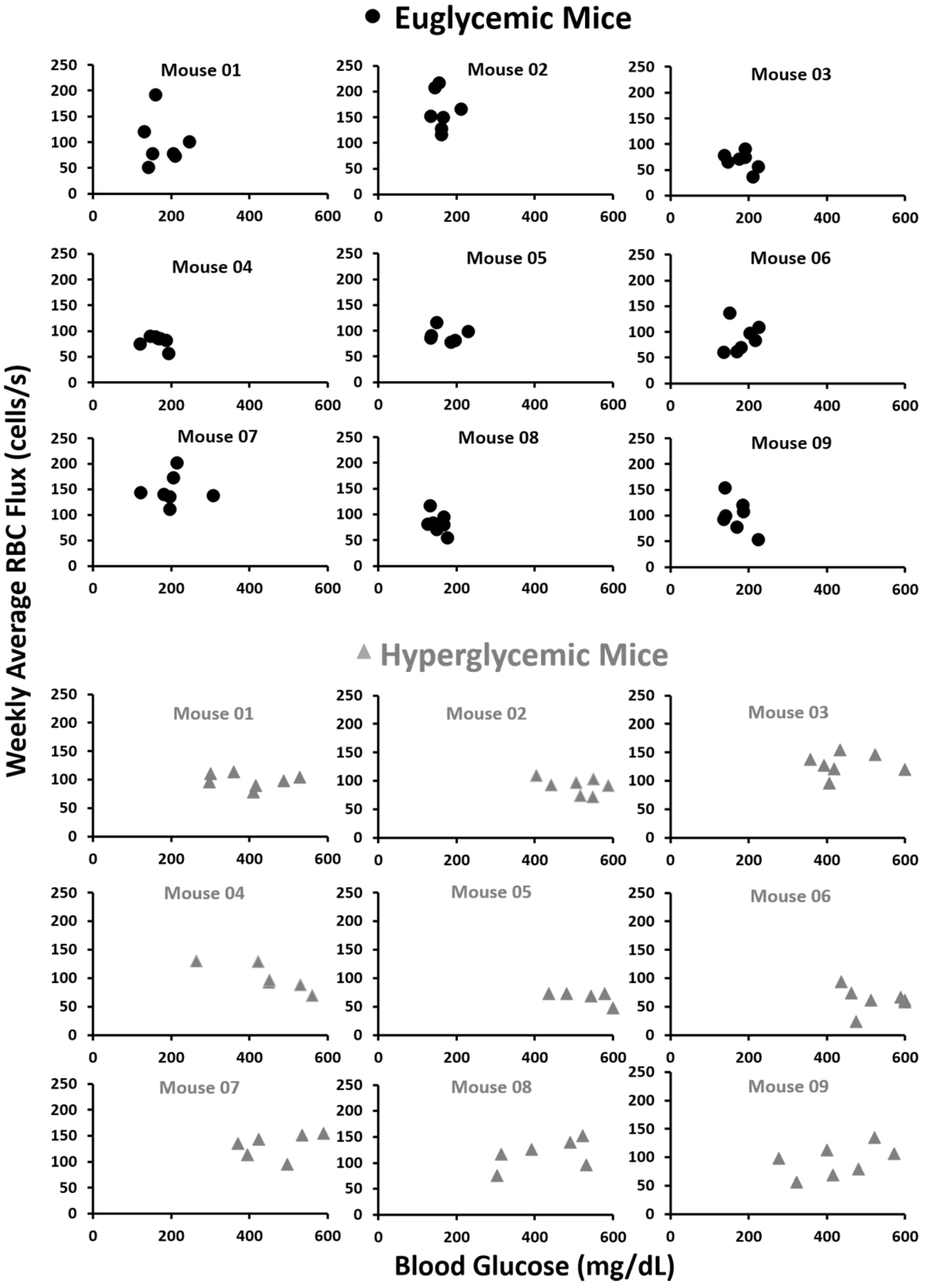

Supplementary figure 6: Effect of increase in blood glucose on weekly average RBC flux for 9 hyperglycemic and 9 euglycemic mice

Weekly average RBC flux for 9 euglycemic mice (black circles) and 9 hyperglycemic mice (gray triangles) as a function of their weekly systemic blood glucose was plotted. For all 18 mice, there is little correlation between weekly RBC flux and systemic blood glucose.

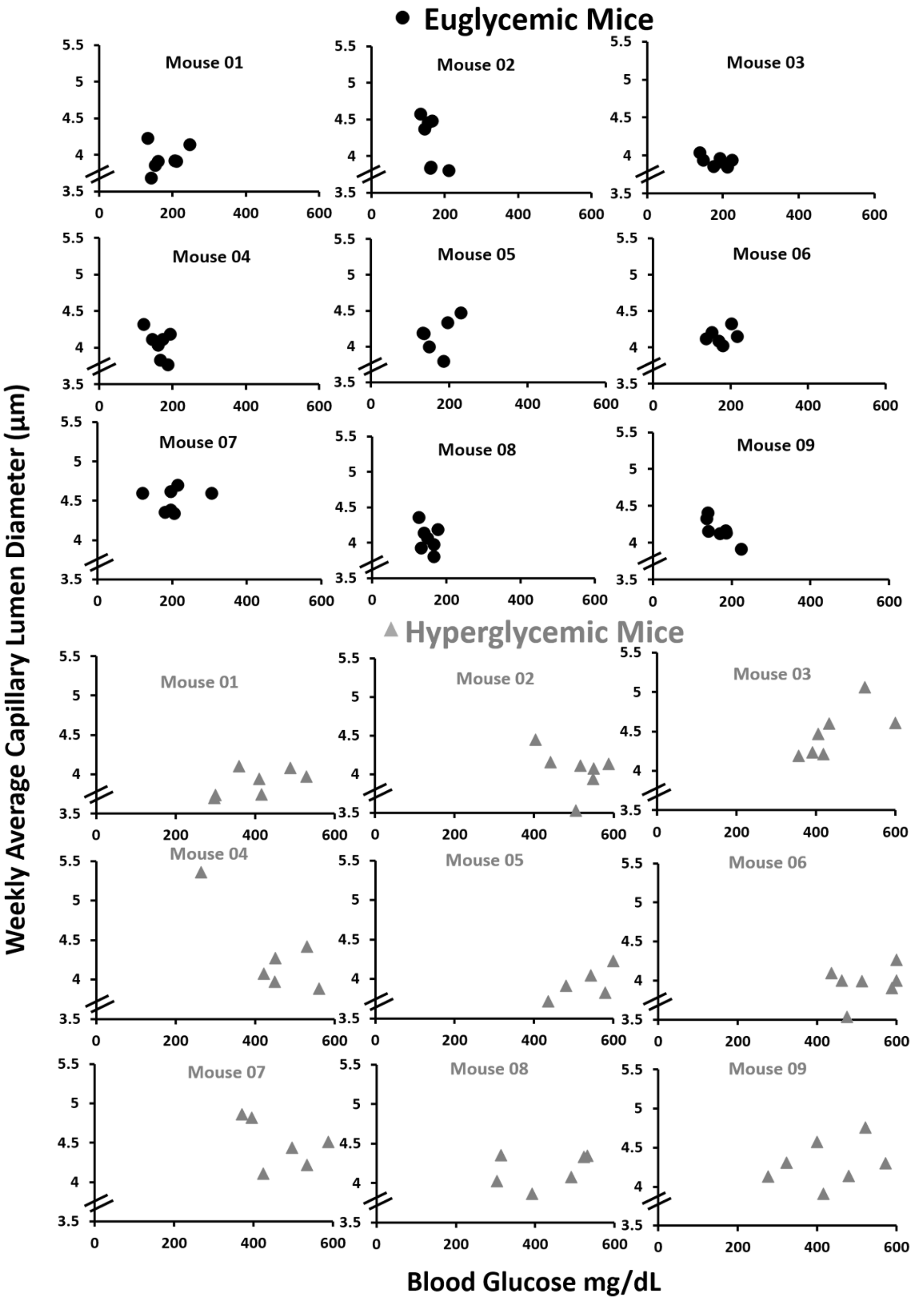

Supplementary figure 7: Effect of increase in blood glucose on weekly average capillary lumen diameter for 9 hyperglycemic and 9 euglycemic mice

Weekly average capillary lumen diameter for 9 euglycemic mice (black circles) and 9 hyperglycemic mice (gray triangles) as a function of their weekly systemic blood glucose was plotted. For all 18 mice, there is little correlation between weekly capillary lumen diameter and systemic blood glucose.

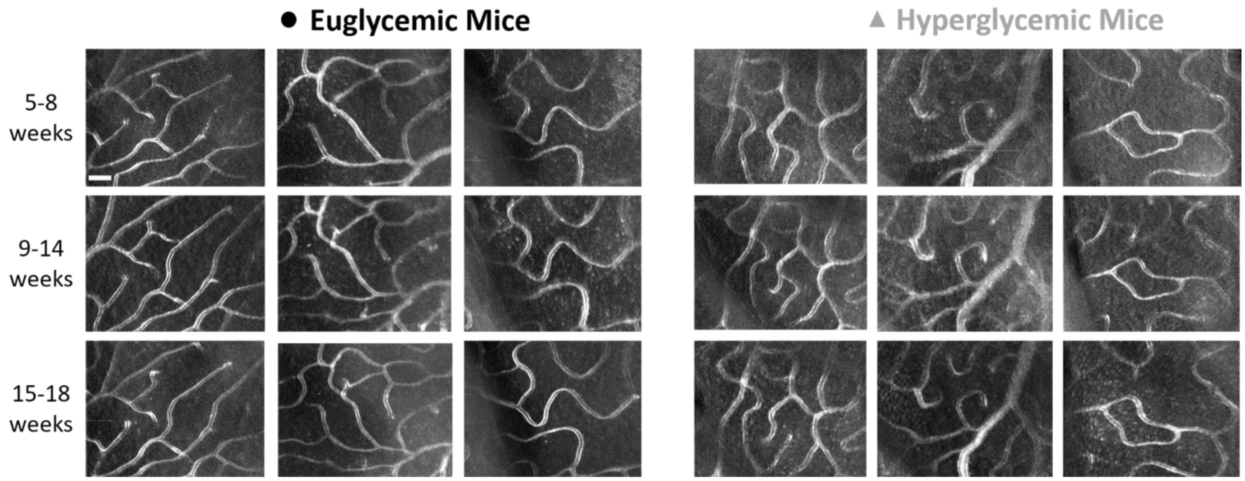

Supplementary figure 8: Capillary anatomy at early, mid and later imaging time points between postnatal week 5-18

Motion contrast images of microvascular structure in the outer plexiform layer in the central retina for euglycemic and hyperglycemic mice (3 samples shown each) at postnatal weeks 5-8, 9-14 and 15-18. Scale bar is 20 µm
